# Topographical Anatomy of the Gluteal and Hamstring Muscles in the Albino Rat (*Rattus norvegicus*)

**DOI:** 10.3390/biology15130986

**Published:** 2026-06-23

**Authors:** Bettina Pretterklieber, Michael L. Pretterklieber

**Affiliations:** Division of Macroscopic and Clinical Anatomy, Gottfried Schatz Research Center, Medical University of Graz, A-8036 Graz, Austria; michael.pretterklieber@medunigraz.at

**Keywords:** albino rat, comparative anatomy, gluteal muscles, hamstring muscles, musculoskeletal system

## Abstract

Background: In comparative anatomy, muscles of the gluteal and hamstring groups are categorized and described inconsistently between, but also within, different species. Due to insufficient information on the exact topography of the individual muscles and a lack of illustrations, it is partly difficult to compare the data provided. Particularly for the albino rat, there is only limited information available for these muscles. The aim of this study was therefore to investigate the presence and morphology of the gluteal and hamstring muscles in the albino rat, and to compare them with other species, including humans. Methods: Both hind limbs of 30 formalin-embalmed male albino rats were carefully dissected. The individual muscles were identified based on their position in relation to nerves and to each other. Therefore, previous descriptions of other species were considered. Results: The gluteus superficialis and tensor fasciae latae muscles formed always a continuous musculoaponeurotic plate. The femorococcygeus and gluteus accessorius muscles were constant structures. The gluteus medius, piriformis and gluteus profundus muscles could always be identified. Within the hamstring group, the two-headed semitendinosus, the one-headed biceps femoris, the semimembranosus, and the caudofemoralis muscles were always present. Conclusions: In this study, a detailed description and dissection guide of the systematic anatomy and topography of the gluteal and hamstring muscles of the albino rat is provided for the first time. The outcomes are intended to help improve knowledge of the anatomy of these muscle groups and to serve as a basis for future studies using rats as an animal model.

## Simple Summary

In comparative anatomy, describing muscles across different species is often inconsistent. For the albino rat, a common laboratory animal, there is even a lack of detailed descriptions and illustrations regarding the muscles of the buttock and back of the thigh. This makes it difficult to compare rat anatomy with that of other species, including humans. Therefore, both hind limbs of 30 albino rats were dissected carefully. The results of this study revealed a consistent muscle pattern. Even though humans walk on two and rats on four legs, the muscular “blueprints” are similar. Some muscles which are large in rats have partly shrunk to remnants in humans due to evolution. These results provide a vital anatomical map that helps scientists better understand anatomy of the albino rat and evolutionary processes.

## 1. Introduction

The muscles of the gluteal and hamstring groups are situated on the outer side of the pelvis and the caudal (posterior) region of the thigh in all mammals, including humans [1,2,3,4,5]. Ontogenetically, the muscles of the gluteal group are derived from the dorsal sacral group, which is innervated by the dorsal sciatic nerve (=common peroneal nerve and gluteal nerves). This group includes the gluteus superficialis (maximus), tensor fasciae latae, femorococcygeus, gluteus medius, piriformis, gluteus profundus (minimus), gluteus accessorius (or gluteus quartus, or scansorius) and tenuissimus muscles [1,6,7]. Phylogenetically, these muscles are derived from the iliofemoralis (abductor femoris) muscle present in more primitive tetrapods [8,9].

On the other hand, the muscles of the hamstring group are derived from the ischiocrural part of the ventral sacral group, which is innervated by the ventral sciatic nerve (=tibial nerve). Due to their location and innervation, the following muscles belong to the ischiocrural (hamstring) muscles: the semitendinosus, semimembranosus, biceps femoris, praesemimembranosus and caudofemoralis muscles [1,6,7]. Phylogenetically, the hamstring muscles are derived from the flexor cruris muscles of more primitive tetrapods [8,9].

Despite this clear classification based on development and, consequently, nerve supply, there are contradictory or inconsistent descriptions and categorisation for some of these muscles. Their names are mixed up or used synonymously. For example—as criticized by some authors [1,8]—the femorococcygeus muscle is confused with muscles of the hamstring group [10,11] or with the tenuissimus muscle [1]. The piriformis and gluteus medius muscles may be completely or partially fused [1,5,9,11]. The gluteus profundus muscle can also consist of two more or less separate parts. Thus, a gluteus accessorius muscle can be present [1,9,12,13]. There also appear to be wide variations in the muscles of the hamstring group and, consequently, in how they are interpreted. In many mammals, the semitendinosus or semimembranosus muscles are said to consist of two heads [1,5,9,11,13,14]. In addition two inconstant muscles of this group, namely the praesemimembranosus and caudofemoralis muscles may occur [8,9]. Some authors described them apparently as parts of other muscles (e.g., the semimembranosus muscle) [5,11,13,14]. The biceps femoris muscle is also described very heterogeneously. According to some authors, it consists of two heads. One originates from the ischial tuber and the second from the sacrum or the cranial coccygeal vertebrae [5,14]. On the other hand, the proper biceps femoris muscle is said to originate in all species exclusively from the ischial tuber (and is only named as ‘biceps’ because of human anatomy) [1,9]. The additional head often mentioned actually corresponds to the femorococcygeus muscle, which belongs to the gluteal group [1,8].

The fact that these muscles are sometimes impossible to compare across species is mainly due to the lack of detailed systematic and topographical anatomical descriptions. The lack of suitable illustrations and photos, as well as a detailed dissection guide, makes uniform naming and identification of these structures difficult. Consequently, there is unfortunately still no uniform nomenclature for comparative anatomy. Although there are some descriptions of these muscle groups in other rodents [15,16,17], only very rudimentary information is available for the albino rat [10,18]. Conversely, authors dealing with comparative anatomy in detail [1,8,9,19] do not address the anatomical features in the albino rat, even though it is frequently used as a common animal model in studies focusing on the musculoskeletal system [20,21,22,23]. Therefore, there is a great need to provide an exact description of these muscle groups in this species. It is particularly important to clarify which muscles of the gluteal and hamstring groups are present in the albino rat, and how they are arranged. A precise topographical description and dissection guide can facilitate identification and harvesting of these muscles in future rat studies. It will also enable experimental interventions on the rat’s musculoskeletal system to be better planned and carried out.

Thus, the aim of this study was to provide an accurate description of the topography of the muscles of the gluteal and hamstring groups in the albino rat, including clear dissection instructions. Therefore, different descriptions for other species should be reviewed and compared to our findings. In addition, we wanted to discuss similarities and differences observed between albino rats and other mammals, particularly humans.

## 2. Materials and Methods

This study is based on careful dissection of both hind limbs of 30 albino rats (60 cases). Due to availability, we used 24 male Sprague-Dawley and 6 male Wistar rats. The animals were sacrificed between 12 and 14 weeks of age after they were used for other studies not involving the hip and thigh regions. Furthermore, they did not undergo experimental procedures altering posture of the limbs or tissue quality. As they were already sacrificed, no further approval of the local animal research ethics committee was necessary for the present study. For these previous studies an institutional ethical approval has been obtained by the Center for Biomedical Research, Medical University of Vienna. The reuse of the specimens followed institutional guidelines.

Immediately after sacrificing, the animals were kept cool and processed for preservation within a few hours. Therefore first, the rats were skinned; the thoracic and abdominal organs, and the external genitals were removed. Then they were embalmed by immersion with a 2% solution of formalin for about four weeks. Subsequently they were stored in a low-percent solution of phenol (approx. 0.5%) until they were dissected. The specimens were not only used for this study, but also for describing other hip and thigh muscles [24,25].

The muscles and nerves of the gluteal and hamstring muscle groups were carefully dissected similarly to how we do it in human specimens. Due to the size of the structures, a magnification lens was used. Nerve branches innervating superficial muscles were marked using coloured yarn (red = superficial perineal nerve, dark blue = caudal gluteal nerve, light blue = cranial gluteal nerve) to trace them back to their origin during further dissection. To visualize and analyze the distal portion and regions of insertion of the medial located hamstring muscles, the adductor muscles had to be dissected and mobilized before, like described recently [24]. The detailed dissection protocol is given as part of the results. Each step was photo-documented using a digital reflex camera (Canon EOS 5D Mark II, Canon Inc., Tokyo, Japan).

**Figure 1:**
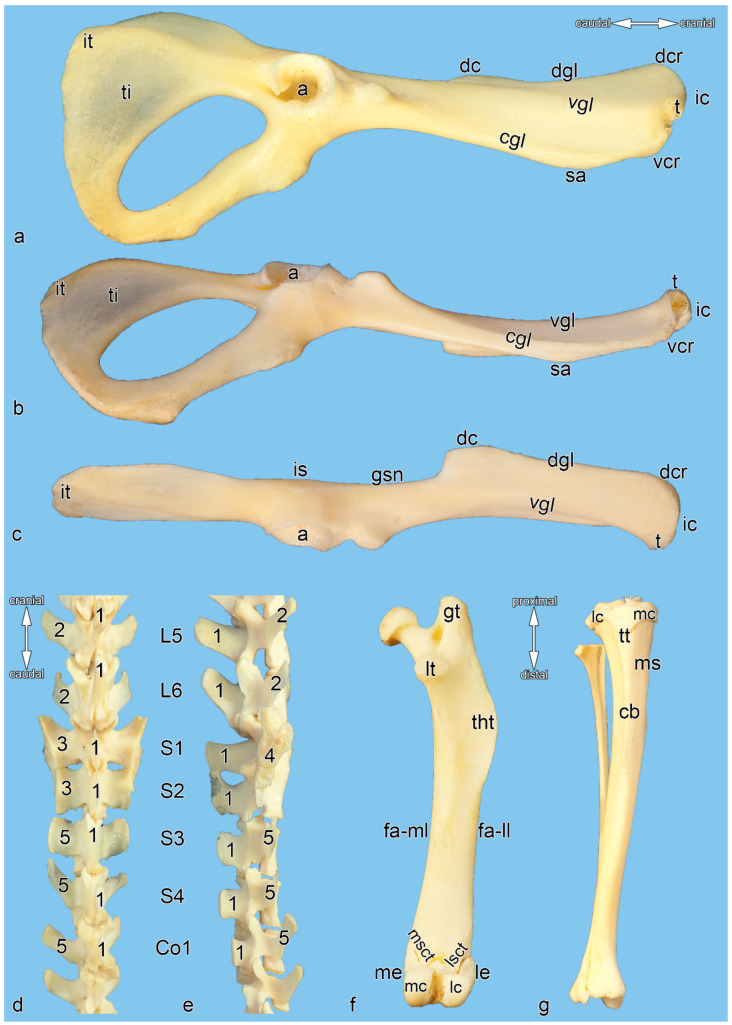
Bony structures important for this study. Photos of macerated bones from a male albino rat to provide an overview of the bony structures important for this study. Isolated right hip bone from the (**a**) lateral, (**b**) ventral, and (**c**) dorsal aspect. a = acetabulum, cgl = caudal gluteal line, dc = dorsal caudal iliac spine, dcr = dorsal cranial iliac spine, dgl = dorsal gluteal line, gsn = greater sciatic notch, ic = iliac crest, is = ischial spine, it = ischial tuber, sa = spina alaris, t = tubercle of iliac crest, ti = tabula of ischium, vcr = ventral cranial iliac spine, vgl = ventral gluteal line. Lumbo-sacral part of the vertebral column from the (**d**) dorsal and (**e**) right aspect. L5–L6 = fifth to sixth lumbar vertebrae, S1–S4 = first to fourth sacral vertebrae, Co1 = first coccygeal vertebra, 1 = spinous process, 2 = costal process, 3 = wing of sacrum, 4 = auricular surface/sacral tuberosity on the wing of sacrum, 5 = transverse process. (**f**) Isolated right femur from the caudal aspect. fa-ml = medial lip of facies aspera, fa-ll = lateral lip of facies aspera, gt = greater trochanter, lc = lateral condyle, le = lateral epicondyle, lsct = lateral supracondylar tuberosity, lt = lesser trochanter, mc = medial condyle, me = medial epicondyle, msct = medial supracondylar tuberosity, tht = third trochanter. (**g**) Isolated right tibia and fibula from the cranial aspect. cb = cranial border of the tibia, mc = medial condyle, ms = medial surface of tibia, lc = lateral condyle, tt = tibial tuberosity.

To visualize bony structures important for this study, macerated bones from one additional male albino rat were photographed (Figure 1). These pictures were also used to mark the areas of origin and insertion of all the muscles examined. All photographs were cropped and labelled using Adobe Photoshop CS6 or Adobe Photoshop 2026 (Adobe Inc., San José, CA, USA) without altering their content.

**Figure 2:**
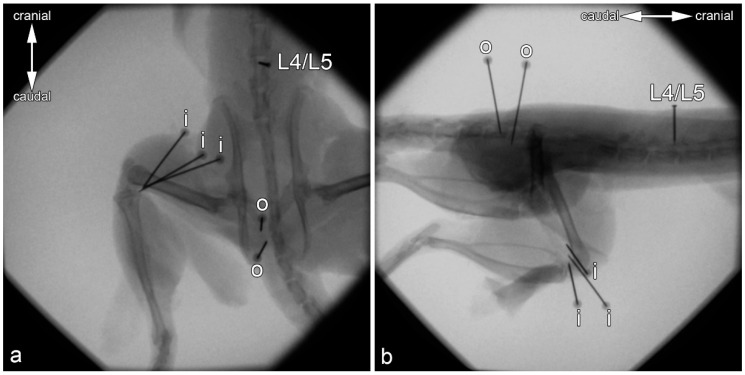
Example of X-rays. The position of the caudofemoralis muscle was marked in an X-ray (**a**) in anterior–posterior view, and (**b**) in lateral view. One pin was placed within the intervertebral disc between the fourth and fifth lumbar vertebrae (L4/L5). Two pins were placed next to the cranial and caudal borders of the muscles origin (o). Three pins were used to mark the borders of the muscles insertion (i).

In addition, X-rays of two cases of each hip muscle were taken using a C-arm (Siemens Siremobil L, Siemens Healthineers AG, Forchheim, Germany) to confirm the exact bony attachments (e.g., exact vertebra) determined during macroscopic examination. As landmark, a pin was inserted into the intervertebral disc between the fourth and fifth lumbar vertebrae. From this point, the vertebrae were counted. The zone of origin and insertion of each individual muscle was marked by inserting pins into the bone. These pins were placed immediately next to the borders of the muscle or tendon. If it was impossible to insert the pin into the bone, the pins were fixed within the soft tissue close to the bone. Each muscle was X-rayed in two planes, in the anterior/posterior-view and in the lateral view. This procedure was helpful especially for superficial muscles. During their dissection, their bony attachments were partly covered. Also, for the deep muscles, this examination was a helpful tool to control the exact points of attachment. Figure 2 shows an example of this procedure.

As the superficial gluteal and tensor fasciae latae muscles formed a common musculoaponeurotic plate, their borders could not exactly be determined by pure macroscopic observation. Thus, at the end of the examination, in six randomly selected cases a whole-mount nerve staining using the modified Sihler’s technique [26,27] was performed. This procedure results in transparent or translucent soft tissue, with the nerve branches being stained. Based on the intramuscular nerve courses, the extent of the two muscle components could be more accurately defined. First the muscles were macerated with a 3% potassium hydroxide (KOH) solution with 0.2 mL of 3% hydrogen peroxide per 100 mL for three weeks to make the tissue transparent. Then, they were decalcified using Sihler’s solution I (1 equivalent glacial acetic acid, 1 equivalent glycerine, 6 equivalents of 1% aqueous chloral hydrate) for almost four weeks. In the next step, the tissue was stained using Sihler’s solution II (1 equivalent Ehrlich’s haematoxylin, 1 equivalent glycerine, 6 equivalents of 1% aqueous chloral hydrate) for one week. Destaining and contrasting were achieved by using Sihler’s solution I again. Following the neutralization process with a 0.05% lithium carbonate solution, the muscles were given into 50% aqueous glycerine for clearing for three days. The muscles were finally stored in a solution of 100% glycerine, adding thymol crystals to serve as an antiseptic agent. The intramuscular course of the nerves was observed under transillumination with a white light transilluminator.

All terms used in this study are following the English version of the *Nomina Anatomica Veterinaria* [28,29]. Only in absence of official terms given by the veterinary anatomy guide, were terms used according to the English version of the *Terminologia Anatomica* [30,31,32]. As some muscles are neither listed in the *Nomina Anatomica Veterinaria* nor in the *Terminologia Anatomica*, they were named according to former authors of comparative anatomy. The term “biceps coxae muscle” used includes the obturator internus and gemellus muscles sharing a common tendon, as recently reported [25].

To discuss the arrangement of the gluteal and hamstring muscles in albino rats compared with that in other species, general comparative and veterinary textbooks, as well as original research, were studied. The comparison with human anatomy is primarily based on detailed human anatomical textbooks and studies concerning the anatomy of the hip muscles, as well as our own years of experience in the dissection room. The respective references are provided alongside the relevant statements in the Section 4.

## 3. Results

The results of this study are based on two different strains. No differences in topography were observed during dissection.

### 3.1. Systematic Anatomy of the Muscles of the Gluteal Group

**Figure 3:**
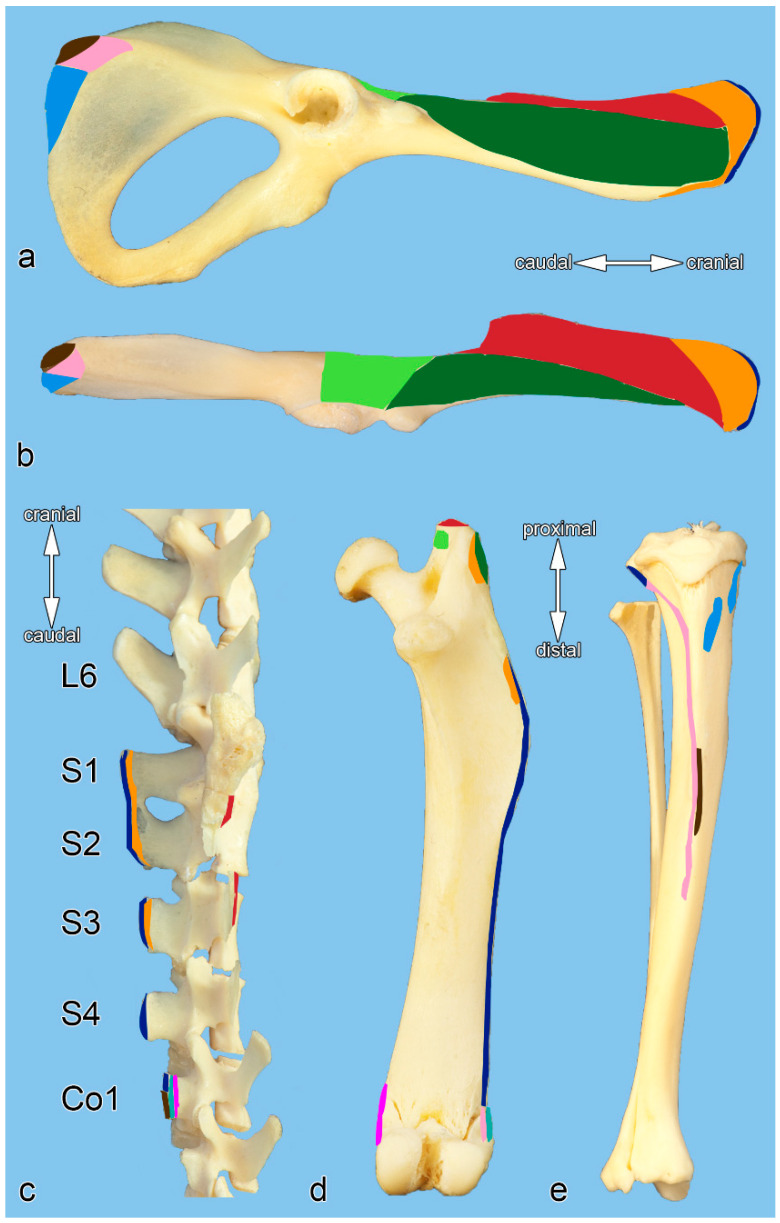
Muscle attachments of the gluteal and hamstring muscles. Origin and insertion areas are colour-coded on photographs of isolated macerated bones from a male albino rat. Isolated right hip bone from the (**a**) lateral and (**b**) dorsal aspect. (**c**) Lumbo-sacral part of the vertebral column from the right aspect. L6 = sixth lumbar vertebra, S1–S4 = first to fourth sacral vertebrae, Co1 = first coccygeal vertebra. (**d**) Isolated right femur from the caudal aspect. (**e**) Isolated right tibia and fibula from the cranial aspect. Colour key: biceps femoris muscle = light pink, caudofemoralis muscle = dark pink, femorococcygeus muscle = turquoise, gluteus accessorius muscle = dark green, gluteus medius muscle = orange, gluteus profundus muscle = light green, gluteus superficialis and tensor fasciae latae muscles = dark blue, piriformis muscle = red, semimembranosus muscle = light blue, semitendinosus muscle = brown.

In all examined albino rats, the following muscles of the gluteal group were constantly present. The systematic details of the investigated muscles are summarized in Table 1. In addition, their areas of origin and insertion are visualized in Figure 3.

#### 3.1.1. Gluteus Superficialis and Tensor Fasciae Latae Muscles

**Figure 4:**
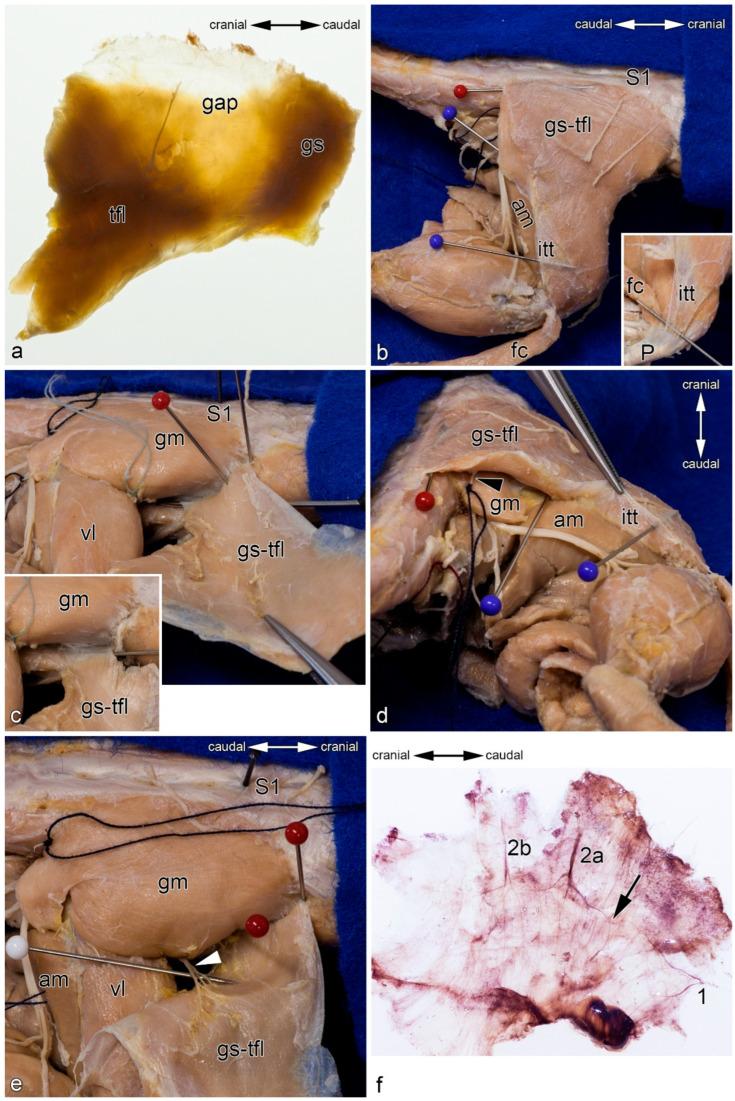
Gluteus superficialis and tensor fasciae latae muscles. (**a**) Deep aspect of a right musculoaponeurotic plate of the gluteus superficialis and tensor fasciae latae musclesunder transillumination. (**b**) The whole complex in situ from the lateral aspect. The red pin was inserted between the gluteus superficialis muscle/the gluteal aponeurosis and the gluteus medius muscle. The blue pins mark its insertion on the third trochanter and the iliotibial tract. The small picture shows the continuation of the iliotibial tract into the lateral patellar retinaculum in detail. (**c**) Origin of the tensor fasciae latae muscle (red pins) from the iliac crest in detail. The small picture shows its deep origin from the aponeurotic septum between the gluteal muscle group and the iliacus muscle. (**d**) Caudal aspect of the gluteus superficialis muscle. The black arrowhead points to the branch of the caudal gluteal nerve innervating the femorococcygeus muscle (already cut through). The blue pins mark the insertion on the third trochanter and the lateral lip of the facies aspera (deep attachment of iliotibial tract). (**e**) Lateral aspect showing the innervation of the tensor fasciae latae muscle by the cranial gluteal nerve (white arrowhead). (**f**) The deep aspect of the musculoaponeurotic plate after the modified Sihler’s procedure under transillumination. The branch from the caudal gluteal nerve (1) ramified intramuscularly in the caudal one quarter to one third. Two branches of the cranial gluteal nerve (2a,2b) supplied the remaining portion of the musculoaponeurotic plate. The black arrow indicates an intramuscular anastomosis between 1 and 2a. In all pictures, the hamstring muscles have been released at least from their origin. dark blue yarn = caudal gluteal nerve, light blue yarn = cranial gluteal nerve, red yarn = superficial perineal nerve, am = adductor minimus and magnus muscles, fc = femorococcygeus muscle, gap = gluteal aponeurosis, gm = gluteus medius muscle, gs = gluteus superficialis muscle, gs-tfl = musculoaponeurotic plate of gluteus superficialis and tensor fasciae latae muscles, itt = iliotibial tract, P = patella/patellar ligament, S1 = spinous process of the first sacral vertebra, tfl = tensor fasciae latae muscle, vl = vastus lateralis muscle.

As the gluteus superficialis and tensor fasciae latae muscles formed a continuous musculoaponeurotic plate (Figure 4a), their description is rolled into one. The triangular cranial muscular portion (i.e., the tensor fasciae latae muscle) and the smaller caudal muscular portion (i.e., the gluteus superficialis muscle) were connected via a thin aponeurosis (i.e., the gluteal aponeurosis in accordance with human terminology [32]). The whole complex was—together with the femorococcygeus muscle—the most superficial muscular layer of the gluteal region. It arose in an aponeurotic manner from the spinous processes of the third and fourth sacral and the first coccygeal vertebrae (Figure 4b). The tensor fasciae latae muscle originated in addition via fleshy fibres from the iliac crest, and from the ventral cranial and dorsal cranial iliac spines. Furthermore, it was attached to the ventral aspect of an aponeurotic septum between the gluteal muscles dorsally, and the iliacus muscle ventrally (Figure 4c). This septum was formed by an aponeurotic origin of the gluteus medius muscle (see below).

The gluteus superficialis muscle inserted partly on the cranial aspect of the third trochanter close to the adductor minimus muscle, which was attached caudally to it. The remaining portion of the gluteus superficialis muscle formed together with the tensor fasciae latae muscle the iliotibial tract. This very thin aponeurotic structure reinforced the lateral patellar retinaculum, which in turn was attached to the lateral femoral and lateral tibial condyles (Figure 4b). The caudal border of the iliotibial tract was attached to the lateral lip of facies aspera starting immediately distal to the third trochanter, forming the lateral femoral intermuscular septum (Figure 4d).

The gluteus superficialis muscle was innervated by the caudal gluteal nerve (Figure 4d), the tensor fasciae latae muscle by two branches of the cranial gluteal nerve (Figure 4e). In the six muscles examined using the modified Sihler’s technique, the intramuscular portion of the caudal gluteal nerve was distributed within the caudal one quarter to one third of the muscular plate. Two branches of the cranial gluteal nerve supplied the remaining part, i.e., the tensor fasciae latae muscle. In addition, in all six muscles an intramuscular anastomosis between the caudal gluteal nerve and the caudal branch of the cranial gluteal nerve seemed to exist (Figure 4f).

#### 3.1.2. Femorococcygeus Muscle

**Figure 5:**
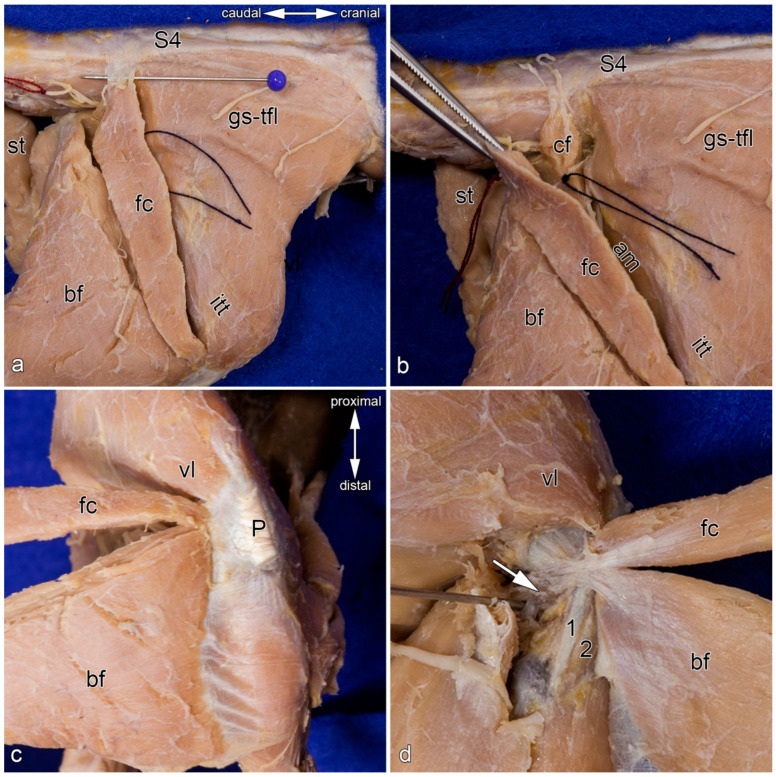
Femorococcygeus muscle. (**a**) Lateral aspect of the hip and thigh regions. Overview of the femorococcygeus muscle. The vertebral head of the semitendinosus muscle has been detached from its origin, as it partly covers the proximal portion of the femorococcygeus muscle. (**b**) The muscle has been detached from its origin to show its innervation by a branch from the caudal gluteal nerve (blue yarn). (**c**) Craniolateral aspect of the knee region. Superficial portion of insertion of the femorococcygeus muscle into the lateral patellar retinaculum between vastus lateralis (proximal) and biceps femoris muscles (distal). (**d**) The femorococcygeus and biceps femoris muscles have been reflected cranially to show the deep portion of insertion of the femorococcygeus muscle on the lateral femoral condyle and the lateral fabella (white arrow). The tendon of insertion covered the proximal attachment of the fibular collateral ligament (1) and—cranial to it—the origin of the extensor digitorum longus muscle (2). The pin shows the origin of the lateral head of the gastrocnemius muscle from the lateral fabella. In all pictures, the vertebral head of the semitendinosus muscle has been released from its origin. am = adductor minimus and magnus muscles, bf = biceps femoris muscle, cf = caudofemoralis muscle, fc = femorococcygeus muscle, itt = iliotibial tract, gs-tfl = musculoaponeurotic plate of gluteus superficialis and tensor fasciae latae muscles, P = patella/patellar ligament, S4 = spinous process of the fourth sacral vertebra, st = semitendinosus muscle (vertebral head), vl = vastus lateralis muscle, red yarn = perineal nerve.

The femorococcygeus muscle was a long, slender muscle, situated in the caudal aspect of the most superficial muscular layer of the gluteal group. It was in close contact to the superficial muscles of the hamstring group. The femorococcygeus muscle originated from the spinous process of the first coccygeal vertebra by means of a thin aponeurosis (Figure 5a). There, it covered the thin aponeurotic origin of the caudofemoralis muscle and seemed to be more or less fused with it (Figure 5b). The femorococcygeus muscle inserted into the lateral patellar retinaculum, and—by a conjoined tendon with the biceps femoris muscle—on the lateral femoral epicondyle and the lateral fabella, reinforcing the iliotibial tract (Figure 5c,d). It was innervated by the caudal gluteal nerve, or sometimes exclusively or additionally by a direct branch from the sacral plexus, which entered the muscle in its proximal portion (Figure 5b).

#### 3.1.3. Gluteus Medius Muscle

**Figure 6:**
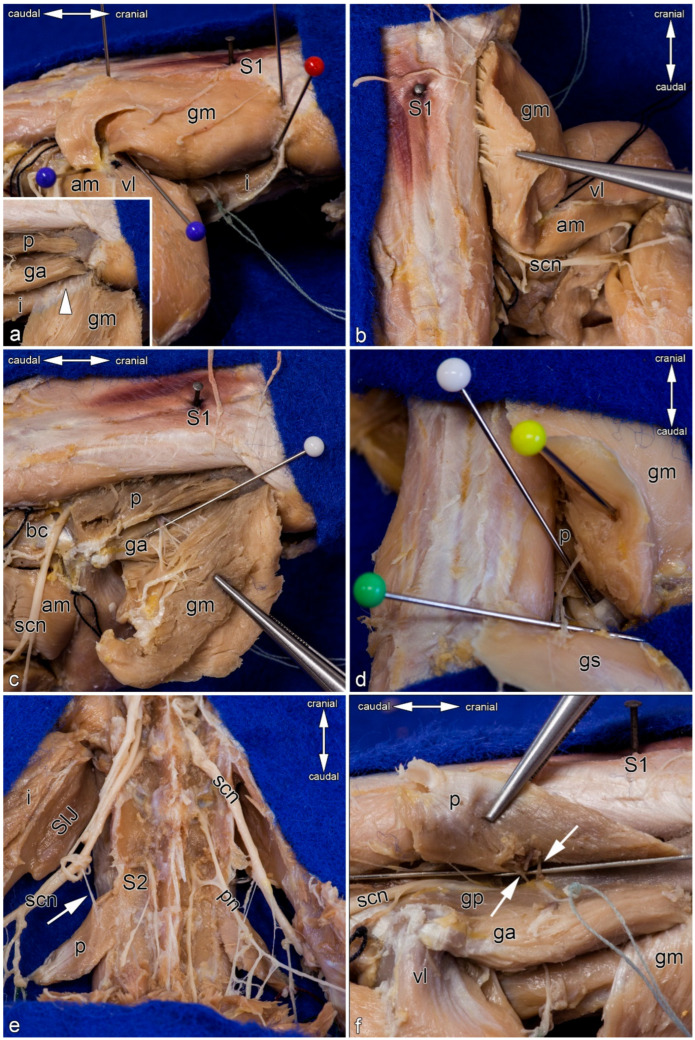
Gluteus medius and piriformis muscles. (**a**) The gluteus medius muscle in situ from the lateral aspect. The small picture shows its deep aponeurotic origin (white arrowhead) in detail after releasing and reflecting the gluteus medius muscle. light blue yarn = branch of cranial gluteal nerve for tensor fasciae latae muscle (cut through). (**b**) Dorsal aspect. The gluteus medius muscle has been released from the spinous processes to show its origin from the fascia covering the muscles of the tail. (**c**) Lateral aspect. During releasing the gluteus medius muscle, its innervation from the cranial gluteal nerve (white pin) gets visible. (**d**) Caudal aspect of the gluteus medius muscle during releasing (yellow pin). The white pin shows the innervation of the caudal portion of the gluteus medius muscle by the caudal gluteal nerve; the green pin shows the innervation of the gluteus superficialis muscle. (**e**) The origin of the piriformis muscle from the second and third sacral vertebrae is shown from the ventral aspect. On the right side, one muscular branch (white arrow) can be observed. Both sacroiliac joints have been luxated, and the psoas major muscle has been removed partially from its origin. (**f**) The piriformis muscle has been released from its insertion to show its innervation (white arrows). Light blue yarn = branch of cranial gluteal nerve for gluteus medius muscle (cut through). In all pictures, the gluteus superficialis, tensor fasciae latae, femorococcygeus, and hamstring muscles have been released at least from their origin. am = adductor minimus and magnus muscles, bc = biceps coxae muscle, ga = gluteus accessorius muscle, gm = gluteus medius muscle, gp = gluteus profundus muscle, gs = gluteus superficialis muscle, i = iliacus muscle, p = piriformis muscle, pn = pudendal nerve, S1 = spinous process of the first sacral vertebra, S2 = caudal part of wing of sacrum, scn = sciatic nerve, SIJ = sacroiliac joint, vl = vastus lateralis muscle.

The gluteus medius muscle was completely covered by the musculoaponeurotic plate formed by the gluteus superficialis and tensor fasciae latae muscles. It was visibly thicker and stronger than this plate. The muscle arose from the spinous processes of the first to third sacral vertebrae, from the deep aspect of the gluteal aponeurosis, from the fascia covering the muscles of the tail, and from the cranial part of the gluteal surface of the ilium between the dorsal and ventral gluteal lines and from the iliac crest. In addition, it originated via an aponeurosis, which was attached to the border of the iliac surface between the ventral cranial iliac spine and the spina alaris. This aponeurosis formed a septum between the gluteal muscles and the origin of the iliacus muscle (Figure 6a,b). The gluteus medius muscle mainly inserted on the caudolateral aspect of the greater trochanter, some deep fibres on the tendon of the piriformis muscle. Its most caudal fibres were attached to the proximal aspect of the third trochanter (Figure 6a). It was innervated by the cranial gluteal nerve (Figure 6c). The most caudal tongue-like portion of the gluteus medius muscle, which was slightly distinct from the rest, received a branch from the caudal gluteal nerve (Figure 6d).

#### 3.1.4. Piriformis Muscle

The piriformis muscle was completely covered by the gluteus medius muscle. Near their origin, these two muscles seemed to be fused. The piriformis muscle was a relatively flat, triangular shaped muscle, which was broad near its origin and narrow towards its insertion (Figure 6c). It originated from the caudal portion of the lateral border of the wing of sacrum just ventral to the sacroiliac joint, and from the transverse process of the third sacral vertebra (Figure 6e). In addition, it was attached to the cranial part of the greater sciatic notch and to the gluteal surface between the dorsal and ventral gluteal lines (caudal to the origin of the gluteus medius muscle). At its craniolateral border, the muscle fibres formed a long tendon, which finally inserted on the tip of the greater trochanter (Figure 6c). Variably, one to three small nerve branches from the sciatic nerve near the origin of the cranial gluteal nerve—or directly from the latter—entered the piriformis muscle on its deep surface (Figure 6f).

#### 3.1.5. Gluteus Accessorius and Gluteus Profundus Muscles

**Figure 7:**
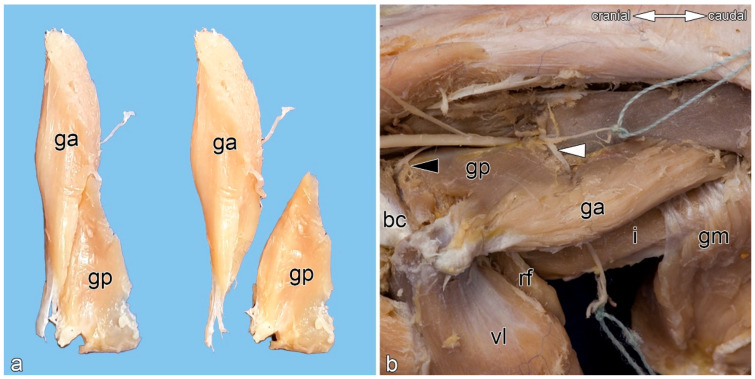
Gluteus accessorius and gluteus profundus muscles. (**a**) The gluteus accessorius and gluteus profundus muscles removed in one part. A gap is visible on their deep side, along which they can be separated from each other. (**b**) The gluteus accessorius and gluteus profundus muscles in situ from the lateral aspect. The cranial gluteal nerve (white arrowhead and light blue yarn) coursed between the two individuals and innervated them. The most caudal portion of the gluteus profundus muscle was innervated by a twig from the nerve to quadratus femoris (black arrowhead). All other muscles of the gluteal group, and the hamstring muscles have been released at least from their origin. bc = biceps coxae muscle, ga = gluteus accessorius muscle, gm = gluteus medius muscle, gp = gluteus profundus muscle, i = iliacus muscle, vl = vastus lateralis muscle, rf = rectus femoris muscle.

The gluteus accessorius and the gluteus profundus muscles formed the deepest layer of the gluteal muscles. In situ, they seemed to be fused. However, it was possible to separate these two individuals after removing them in one part (Figure 7a).

The spindle-shaped gluteus accessorius muscle arose from the gluteal surface between the ventral and caudal gluteal lines. Deep fibres originated from the intermuscular septum formed by the deep origin of the gluteus medius muscle. It inserted on the craniolateral aspect of the greater trochanter between the insertion of the piriformis muscle on the tip and the origin of the vastus lateralis muscle from the lateral aspect of the greater trochanter. The gluteus accessorius muscle was innervated by the cranial gluteal nerve (Figure 7b).

The fan-shaped gluteus profundus muscle arose from the ischial spine cranial to the gemellus muscle and the greater sciatic notch and reached the dorsal border of the acetabulum. It reinforced the hip joint capsule and inserted on the medial aspect of the greater trochanter dorsocranial to the tendon of the biceps coxae muscle. The cranial portion of the gluteus profundus muscle was innervated by the cranial gluteal nerve. Its most caudal fibres were innervated by the nerve to quadratus femoris muscle from the sciatic nerve (Figure 7b).

### 3.2. Systematic Anatomy of the Muscles of the Hamstring Group

In all examined albino rats, the following muscles of the hamstring group were constantly present. The systematic details of the investigated muscles are summarized in Table 2. In addition, their areas of origin and insertion are visualized in Figure 3.

#### 3.2.1. Semitendinosus Muscle

**Figure 8:**
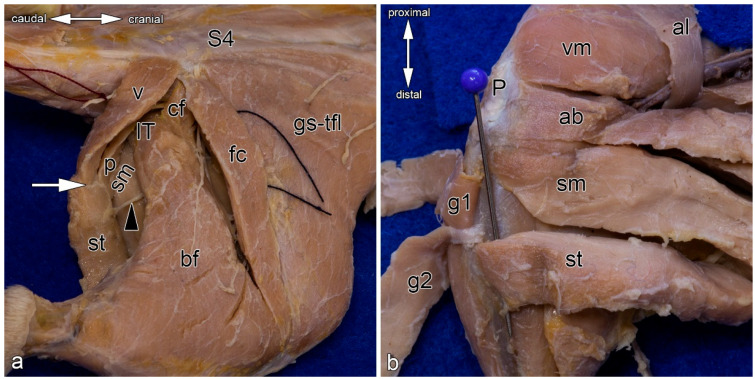
Semitendinosus muscle. (**a**) The semitendinosus muscle in situ from the lateral aspect of the hip and thigh. The tendinous intersection (white arrow) is visible. Deep to its vertebral head, the superficial perineal nerve (red yarn) courses. A branch from the hamstring muscular branch (black arrowhead) innervated its pelvic head. (**b**) On the medial aspect of the knee region, the insertion of the semitendinosus muscle is visible. The cranial and caudal gracilis muscles, and the adductor longus muscle have been released from their origin. ab = adductor brevis muscle, al = adductor longus muscle, bf = biceps femoris muscle, cf = caudofemoralis muscle, fc = femorococcygeus muscle, g1 = cranial gracilis muscle, g2 = caudal gracilis muscle, gs-tfl = musculoaponeurotic plate of gluteus superficialis and tensor fasciae latae muscles, IT = ischial tuber, P = patella/patellar ligament, p = pelvic head of semitendinosus muscle, S4 = spinous process of the fourth sacral vertebra, sm = semimembranosus muscle, st = semitendinosus muscle, v = vertebral head of semitendinosus muscle, vm = vastus medialis muscle.

The semitendinosus muscle was the most caudal structure in the lateral aspect of the thigh, bending into the medial aspect of the thigh. It originated by two heads. The vertebral head originated via an aponeurosis from the spinous processes of the first two coccygeal vertebrae. The pelvic head originated from the dorsocaudal aspect of the ischial tuber next to the biceps femoris muscle. Both heads of the semitendinosus muscle united to form a tendinous intersection on the deep aspect of the muscle (Figure 8a). This intersection also served as attachment for the biceps femoris muscle (see below). After the union of the two heads, the semitendinosus muscle turned around to reach the medial aspect of the thigh. There it inserted caudally to the caudal gracilis muscle on the cranial border of the tibia (Figure 8b). Between the deep aspect of the muscle and the medial surface of the tibia, a bursa was situated. The vertebral head was almost always innervated by the superficial perineal nerve, or occasionally by the hamstring muscular branch of the tibial nerve. The latter innervated constantly the pelvic head (Figure 8a). Sometimes, additional branches from the tibial nerve itself entered the pelvic head too.

#### 3.2.2. Biceps Femoris Muscle

**Figure 9:**
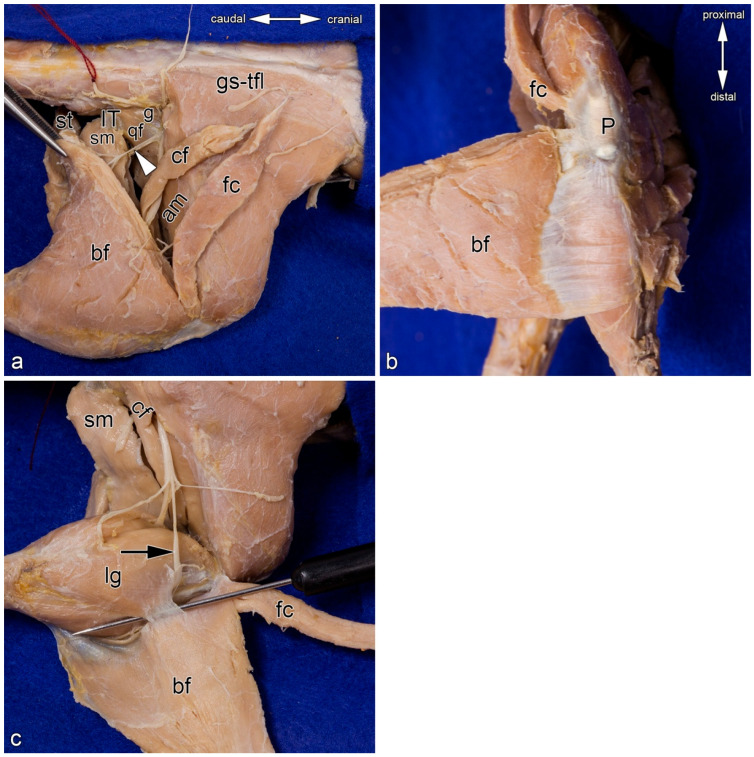
Biceps femoris muscle. (**a**) The biceps femoris muscle in situ from the lateral aspect of the hip and thigh. Its origin has been released, so its innervation from the hamstring muscular branch (white arrowhead) was visible. (**b**) On the cranial aspect of the leg, the superficial portion of insertion of the biceps femoris muscle into the lateral patellar retinaculum and on the cranial border of the tibia is visible. (**c**) During reflecting the biceps femoris muscle, the aponeurotic connection with the lateral head of the gastrocnemius muscle (shown with the probe) becomes visible. Just proximal to it, the common peroneal nerve (black arrow) disappeared. In all pictures the femorococcygeus, semitendinosus and caudofemoralis muscles have been released from their origin. am = adductor minimus and magnus muscles, bf = biceps femoris muscle, cf = caudofemoralis muscle, fc = femorococcygeus muscle, g = gemellus muscle, gs-tfl = musculoaponeurotic plate of gluteus superficialis and tensor fasciae latae muscles, IT = ischial tuber, lg = lateral head of gastrocnemius muscle, P = patella/patellar ligament, qf = quadratus femoris muscle, sm = semimembranosus muscle, st = semitendinosus muscle.

The biceps femoris muscle was a broad triangular formed muscle, narrow near its origin and broad near its insertion. It occupied most of the superficial layer in the lateral aspect of the thigh. It originated from the dorsocranial aspect of the ischial tuber (Figure 8a and Figure 9a). It was also attached to the tendinous intersection on the inner aspect of the semitendinosus muscle described above. The biceps femoris muscle reinforced the lateral patellar retinaculum. By means of a broad aponeurosis, it inserted on the proximal half to two thirds of the cranial border of the tibia (Figure 9b). In addition, deep fibres inserted together with the femorococcygeus muscle on the lateral femoral epicondyle and the lateral fabella (Figure 5d). The remaining deep fibres inserted on the lateral tibial condyle. Distal to the course of the common peroneal nerve towards the peroneal compartment, constantly, an aponeurosis connected the lateral head of the gastrocnemius muscle with the inner aspect of the biceps femoris muscle (Figure 9c). The biceps femoris muscle was innervated by two to three small twigs from the hamstring muscular branch of the tibial nerve (Figure 9a).

#### 3.2.3. Semimembranosus Muscle

**Figure 10:**
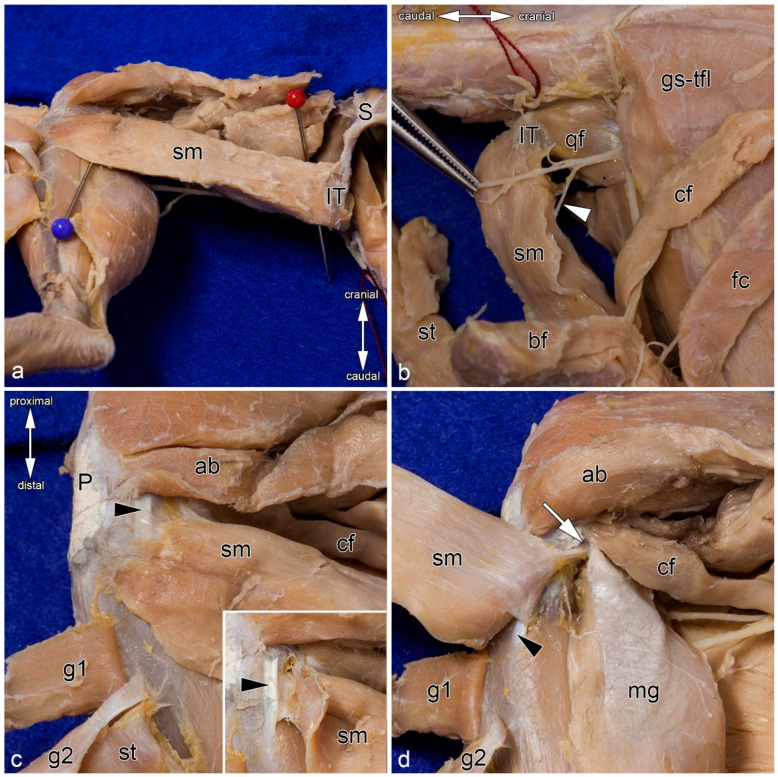
Semimembranosus muscle. (**a**) The position of the semimembranosus muscle from the medial aspect of the thigh. (**b**) Lateral view. Its origin from the ventral aspect of the ischial tuber and its innervation (white arrowhead) from the hamstring muscular branch are shown. (**c**) The superficial portion of its insertion into the medial patellar retinaculum and on the medial tibial condyle is shown from the medial aspect of the thigh. In the small picture, the superficial portion of its insertion has been released. Its proximity to the tibial collateral ligament (black arrowhead) and its deep portion of insertion are visible. (**d**) The muscle has been detached from its origin and reflected towards its insertion to show its attachment on the medial fabella (white arrow). In all pictures, the femorococcygeus, all other hamstring, and both gracilis muscles have been released from their origin. ab = adductor brevis muscle, bf = biceps femoris muscle, cf = caudofemoralis muscle, fc = femorococcygeus muscle, g1 = cranial gracilis muscle, g2 = caudal gracilis muscle, gs-tfl = musculoaponeurotic plate of gluteus superficialis and tensor fasciae latae muscles, IT = ischial tuber, mg = medial head of gastrocnemius muscle, P = patella/patellar ligament, qf = quadratus femoris muscle, S = pelvic symphysis, sm = semimembranosus muscle, st = semitendinosus muscle.

The semimembranosus muscle was a huge and long muscle coursing from the lateral to the medial aspect of the thigh (Figure 10a). Both, its proximal and distal portions were covered by other muscles. It originated deep to the semitendinosus and biceps femoris muscles from the ventral aspect of the ischial tuber, and from the caudal margin of the tabula of ischium (Figure 10b). Subsequently, it bended around into the medial aspect of the thigh where it divided into a superficial and a deep portion with the tibial collateral ligament in between. The superficial portion inserted on the medial tibial condyle and the medial surface of the tibia, just medial to the patellar ligament, and reinforced the medial patellar retinaculum (Figure 10c). Between the muscle and the medial tibial condyle, and the medial collateral ligament a bursa was established. The deep portion was also attached to the medial tibial condyle (again with a bursa) but deep to the tibial collateral ligament. In addition, it inserted on the distomedial aspect of the medial fabella (Figure 10d). There, it seemed to reinforce the caudomedial part of the knee joint capsule. The semimembranosus muscle was innervated by the hamstring muscular branch of the tibial nerve (Figure 10b).

#### 3.2.4. Caudofemoralis Muscle

**Figure 11:**
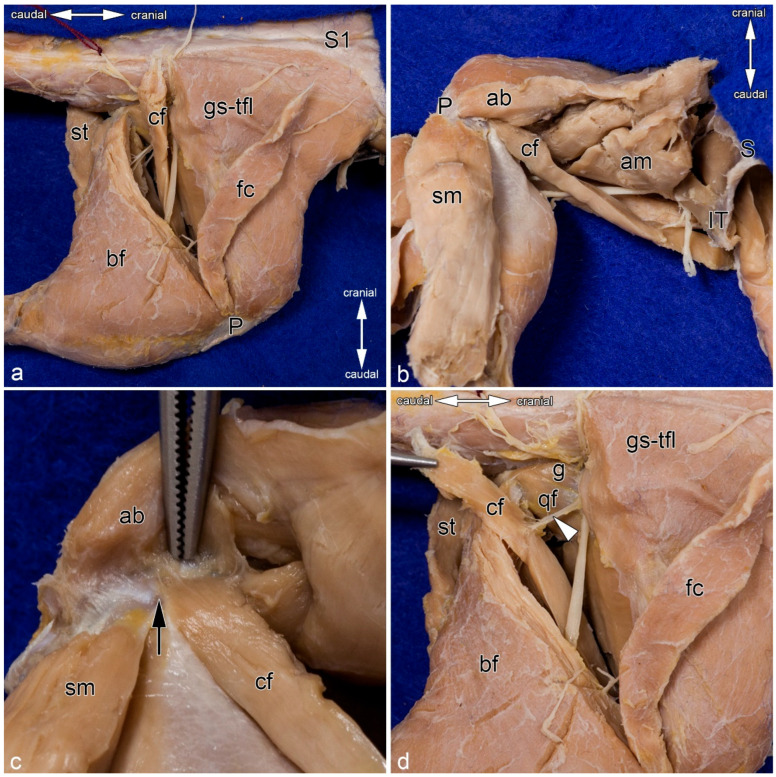
Caudofemoralis muscle. (**a**) The origin and position of the caudofemoralis muscle in situ from the lateral aspect of the thigh. (**b**) Its position and insertion on the medial femoral epicondyle and medial supracondylar tuberosity from the medial aspect of the thigh. (**c**) Its insertion on the medial fabella (black arrow). (**d**) It has been mobilized from its origin to show its innervation (white arrowhead) from the hamstring muscular branch. In all pictures, the femorococcygeus muscle, partly the other hamstring muscles, and all adductor muscles have been detached from their origin. ab = adductor brevis muscle, am = adductor minimus and magnus muscles, bf = biceps femoris muscle, cf = caudofemoralis muscle, fc = femorococcygeus muscle, g = gemellus muscle, gs-tfl = musculoaponeurotic plate of gluteus superficialis and tensor fasciae latae muscles, IT = ischial tuber, P = patella/patellar ligament, qf = quadratus femoris muscle, S = pelvic symphysis, S1 = spinous process of the first sacral vertebra, sm = semimembranosus muscle, st = semitendinosus muscle.

The caudofemoralis muscle was a long and slender muscle. Its proximal portion was situated in the lateral aspect of the thigh, deep to the femorococcygeus muscle and cranially to the semitendinosus muscle. It originated by a thin aponeurosis from the spinous process of the first coccygeal vertebra (Figure 11a). It inserted deep to the genicular part of the adductor brevis muscle on the medial femoral epicondyle and on the medial supracondylar tuberosity. It was also attached to the proximomedial aspect of the medial fabella (Figure 11b,c). It was innervated by the hamstring muscular branch (Figure 11d), or occasionally via a direct muscular branch of the tibial nerve. In some instances, its proximal portion received an additional twig from the muscular branch for the femorococcygeus muscle, or from that innervating the vertebral head of the semitendinosus muscle.

### 3.3. Topography and Dissection Guide

**Figure 12:**
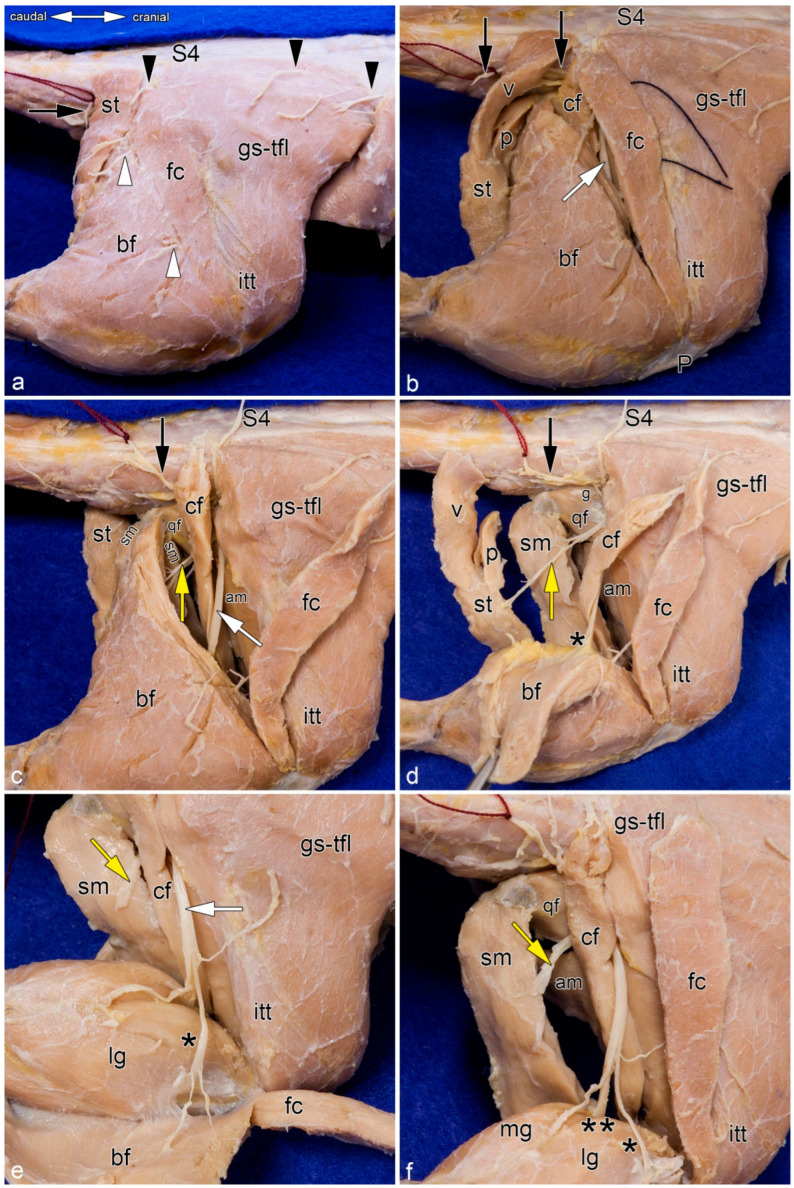
Topography of the hamstring muscles. (**a**) Lateral aspect of the hip and thigh, subcutaneous layer. (**b**) The fascia lata has been removed, and the hamstring muscles have been separated in situ. (**c**) Both heads of the semitendinosus muscle, and the femorococcygeus muscle have been released from their origin. (**d**) The biceps femoris muscle has been released from its origin, exposing the common peroneal nerve. (**e**) The aponeurotic connection between the inner aspect of the biceps femoris muscle and the lateral head of the gastrocnemius muscle. (**f**) Subdivision of the sciatic nerve and topographic relation between the caudofemoralis muscle and this nerve as well as the hamstring muscular branch. For details refer to the text. Black arrowheads = cranial, middle, and caudal clunial nerves, white arrowheads = caudal cutaneous femoral nerve (proximal) and lateral cutaneous sural nerve (distal), black arrow and red yarn = superficial perineal nerve, white arrow = sciatic nerve, yellow arrow = hamstring muscular branch, asterisk = common peroneal nerve, double asterisks = tibial nerve, am = adductor minimus and magnus muscles, bf = biceps femoris muscle, cf = caudofemoralis muscle, fc = femorococcygeus muscle, g = gemellus muscle, gs-tfl = musculoaponeurotic plate of gluteus superficialis and tensor fasciae latae muscles, itt = iliotibial tract, lg = lateral head of gastrocnemius muscle, mg = medial head of gastrocnemius muscle, p = pelvic head of semitendinosus muscle, qf = quadratus femoris muscle, S4 = spinous process of the fourth sacral vertebra, sm = semimembranosus muscle, st = semitendinosus muscle, v = vertebral head of semitendinosus muscle.

After removing skin and subcutaneous tissue, the superficial muscles were already visible through the fascia lata. Almost the whole gluteal region and the lateral region of the thigh were covered by the musculoaponeurotic plate of the gluteus superficialis and tensor fasciae latae muscles, and the superficial portion of the iliotibial tract. The femorococcygeus muscle was situated next to the gluteus superficialis muscle, partly covering its caudal border. The most proximal portion of the femorococcygeus muscle was covered by the vertebral head of the semitendinosus muscle. Between these two diverging muscles the triangular shaped biceps femoris muscle was situated. Superficial to the tensor fasciae latae muscle, the cranial clunial nerves coursed, coming from the lumbar region. On the level of the sacral vertebrae, the middle clunial nerves were visible. On the caudal border of the gluteus superficialis muscle, the caudal clunial nerves pierced the fascia. Between the femorococcygeus and biceps femoris muscles, the caudal cutaneous femoral nerve, and the lateral cutaneous sural nerve emerged. On the caudal border of the vertebral head of the semitendinosus muscle the superficial perineal nerve appeared. As the external genitals and skin in this region had already been removed prior to embalming, it was not possible to completely preserve its entire course. All these nerves were helpful to separate the individual muscles by blunt dissection, and to remove the fascia lata (Figure 12a).

First, the semitendinosus and femorococcygeus muscles were mobilized. The latter was in close relationship with the cranial border of the biceps femoris muscle, the caudal border of the gluteus superficialis muscle, and the iliotibial tract. In doing so, the iliotibial tract, its deep portion (i.e., the lateral femoral intermuscular septum), and the innervation of the femorococcygeus muscle were preserved (Figure 12b). This fine nerve originated from the caudal gluteal nerve and appeared on the caudal border of the gluteus superficialis muscle. It entered the deep aspect of the proximal portion of the femorococcygeus muscle (Figure 5b). Deep to the femorococcygeus muscle, a small portion of the sciatic nerve was already visible at the cranial border of the biceps femoris muscle. When mobilizing the vertebral head of the semitendinosus muscle, care had to be taken of its innervation from the superficial perineal nerve. This nerve was situated close to the lateral aspect of the tail and deep to the proximal portion of the caudofemoralis muscle. On the caudal border of the latter, the superficial perineal nerve reached the deep surface of the vertebral head of the semitendinosus muscle. Now, the pelvic head of the semitendinosus muscle and the origin of the caudofemoralis muscles became visible. At the level of the union of the two heads of the semitendinosus muscle, a small tendinous intersection was established on the inner aspect of the muscle. The caudal border of the biceps femoris muscle was also connected to this intersection and had to be released carefully. Now the origin of the biceps femoris muscle and part of the semimembranosus muscle were uncovered (Figure 12b). Between these two muscles, the twig from the hamstring muscular branch innervating the pelvic head of the semitendinosus muscle appeared (Figure 8a). The hamstring muscular branch itself originated from the medial portion of the sciatic nerve (i.e., the tibial nerve), and coursed between the caudofemoralis muscle (lateral), and the gemellus and quadratus femoris muscles (medial) to reach the gap between the biceps femoris and semimembranosus muscles (Figure 12b,c).

Both heads of the semitendinosus muscle were released from their origin on the spinous processes of the first two coccygeal vertebrae and the dorsocaudal aspect of ischial tuber, respectively. To reflect the whole muscle towards its insertion on the cranial border of the tibia, the innervation of both heads had to be cut through.

The femorococcygeus muscle was also detached from the spinous process of the first coccygeal vertebra. Its thin tendon of origin was in close contact with the thin tendon of origin of the caudofemoralis muscle; the latter should not be harmed (Figure 12c). During reflecting the femorococcygeus muscle towards its common insertion with the adjacent portion of the biceps femoris muscle, its innervation from the caudal gluteal nerve had to be cut through (Figure 5b). Deep to the proximal portion of the femorococcygeus muscle, the sciatic nerve coursed distally and disappeared between the biceps femoris muscle (superficial) and the caudofemoralis muscle (deep). This nerve was also in close contact to the gemellus and quadratus femoris muscles, and to the insertion of the adductor minimus muscle. As described above, deep to the caudofemoralis muscle, the hamstring muscular branch coursed in a caudodistal direction (Figure 12c,d).

The next step was to detach the origin of the biceps femoris muscle from the dorsocranial aspect of the ischial tuber and to reflect it towards its insertion. The origin of the muscle was between the already released pelvic head of the semitendinosus muscle caudally and the quadratus femoris muscle cranially (Figure 12d). Thereby, its innervation from the hamstring muscular branch had to be cut through (Figure 9a). Near the insertion of the biceps femoris muscle, one had to pay attention on the common peroneal nerve. The latter gave off the lateral cutaneous sural nerve and coursed into the peroneal compartment of the leg. An aponeurotic connection between the inner aspect of the biceps femoris muscle and the lateral head of the gastrocnemius muscle just distal to the entrance of the common peroneal nerve into the peroneal compartment was now visible (Figure 12e). It had to be released to get full access to the deep portion of insertion of the biceps femoris muscle. This portion inserted together with the femorococcygeus muscle on the lateral femoral epicondyle and on the lateral fabella. The remaining deep fibres inserted on the lateral tibial condyle. Between these two bony attachments, a tendinous arc was established, crossing superficial to the fibular collateral ligament and the tendon of the extensor digitorum muscle (Figure 5d).

Now the origin and proximal portions of the caudofemoralis and semimembranosus muscles were completely visible (Figure 12f). The semimembranosus muscle originated from the ventral aspect of the ischial tuber (in close contact with the quadratus femoris muscle). Ventrally to the semimembranosus muscle, the caudal gracilis muscle as well as the adductor magnus and minimus muscles arose. With its deep portion, the caudofemoralis muscle covered the gemellus and quadratus femoris muscles (Figure 12b). As mentioned above, the caudofemoralis muscle separated the hamstring muscular branch from the sciatic nerve. The sciatic nerve subdivided into the common peroneal nerve and the tibial nerve; the latter gave off the caudal cutaneous sural nerve and disappeared between the heads of the gastrocnemius muscle (Figure 12f).

As the last step concerning the hamstring muscles, the caudofemoralis and semimembranosus muscles were detached from their origin, and their innervation from the hamstring muscular branch was cut through. Now, the semitendinosus, semimembranosus and caudofemoralis muscles were reflected towards their insertion in the medial aspect of the thigh. To fully visualize their insertion, the adductor muscles must first be reflected or partially released (Figure 8b, Figure 10a,c,d and Figure 11b,c). The precise procedure was recently published [24]. In the medial aspect of the thigh, the semitendinosus muscle was situated distally to the adductor muscles and the semimembranosus muscle. It was partly covered by the caudal gracilis muscle, which inserted immediately cranial to the semitendinosus muscle (Figure 8b). The semimembranosus muscle was covered by the cranial and caudal gracilis muscles, and partly by the genicular part of the adductor brevis muscle. It was also closely related to the caudofemoralis muscle, which inserted just proximal to it (Figure 10a,c,d). The caudofemoralis muscle was also covered by the genicular part of the adductor brevis muscle (Figure 11b,c). It defined the lateral border of the adductor hiatus, through which the femoral vessels coursed to reach the popliteal fossa [27]. If one wants to harvest the hamstring muscles, they can now be detached from their respective insertions.

**Figure 13:**
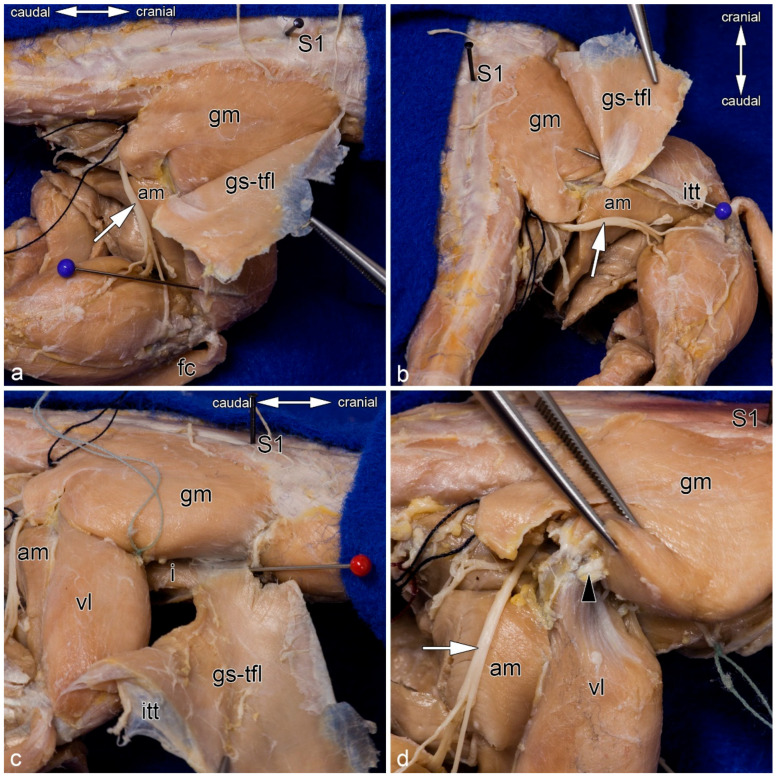
Topography of the gluteal muscles 1. (**a**) Dorsolateral aspect. Exposing gluteus medius muscle. (**b**) Its insertion on the third trochanter. (**c**) Origin of the tensor fasciae latae muscle from the septum between the gluteal muscles and the iliac muscle. (**d**) After detaching the gluteus medius muscle from the proximal aspect of the third trochanter and elevating its main portion the tendon of the gluteus accessorius muscle (black arrowhead) is visible. For details refer to the text. white arrow = sciatic nerve, dark blue yarn = caudal gluteal nerve, light blue yarn = cranial gluteal nerve, am = adductor minimus and magnus muscles, fc = femorococcygeus muscle, gm = gluteus medius muscle, gs-tfl = musculoaponeurotic plate of gluteus superficialis and tensor fasciae latae muscles, i = iliacus muscle, itt = iliotibial tract, S1 = spinous process of the first sacral vertebra, vl = vastus lateralis muscle.

Detaching the muscles of the hamstring group enabled the access to the muscles of the gluteal group. Deep to the musculoaponeurotic plate of the gluteus superficialis and tensor fasciae latae muscles, the gluteus medius muscle was situated (Figure 13a). It was already visible on the caudal border of the gluteus superficialis muscle (Figure 4d). These two muscular layers were separated starting near their insertion on the third trochanter using the course of the caudal gluteal nerve. This nerve appeared on the caudal border of the gluteus medius muscle, where it gave off the branch for the femorococcygeus muscle, which has been already cut through. Then, it curved around the gluteus medius muscle to reach the deep aspect of the gluteus superficialis muscle (Figure 4d). The gluteus superficialis and tensor fasciae latae muscles were detached from the spinous processes of the first coccygeal and all sacral vertebrae in a caudocranial direction. Thereby, the innervation of the gluteus superficialis muscle from the caudal gluteal nerve was cut through (Figure 13a). Only now, the insertion of the gluteus superficialis muscle on the cranial aspect of the third trochanter became fully visible. Next to it, the most caudal fibres of the gluteus medius muscle, the adductor minimus muscle, and the cranial portion of the adductor maximus muscle were attached on the caudal aspect of the third trochanter (Figure 13b). The gluteus superficialis muscle was released from the third trochanter, and the iliotibial tract from its attachment on the lateral lip of the facies aspera. Thereby the vastus lateralis muscle became visible. It was attached cranial to the gluteus superficialis muscle and the deep portion of the iliotibial tract (Figure 13c). During further reflecting the gluteus superficialis and tensor fasciae latae muscles, the cranial gluteal nerve became visible. This nerve appeared on the ventrolateral border of the gluteus medius muscle, caudal to the deep origin of the tensor fasciae latae muscle (Figure 4e).

The innervation of the tensor fasciae latae muscle was cut through, and the muscle was detached from its origin on the iliac crest. Thus, its deep aponeurotic origin from the septum between the gluteal muscles and the iliacus muscle became visible (Figure 13c). This septum was formed by an aponeurotic attachment of the gluteus medius muscle (see below). Finally, the attachment on this septum was released to reflect the entire musculoaponeurotic plate together with the iliotibial tract towards the lateral patellar retinaculum.

**Figure 14:**
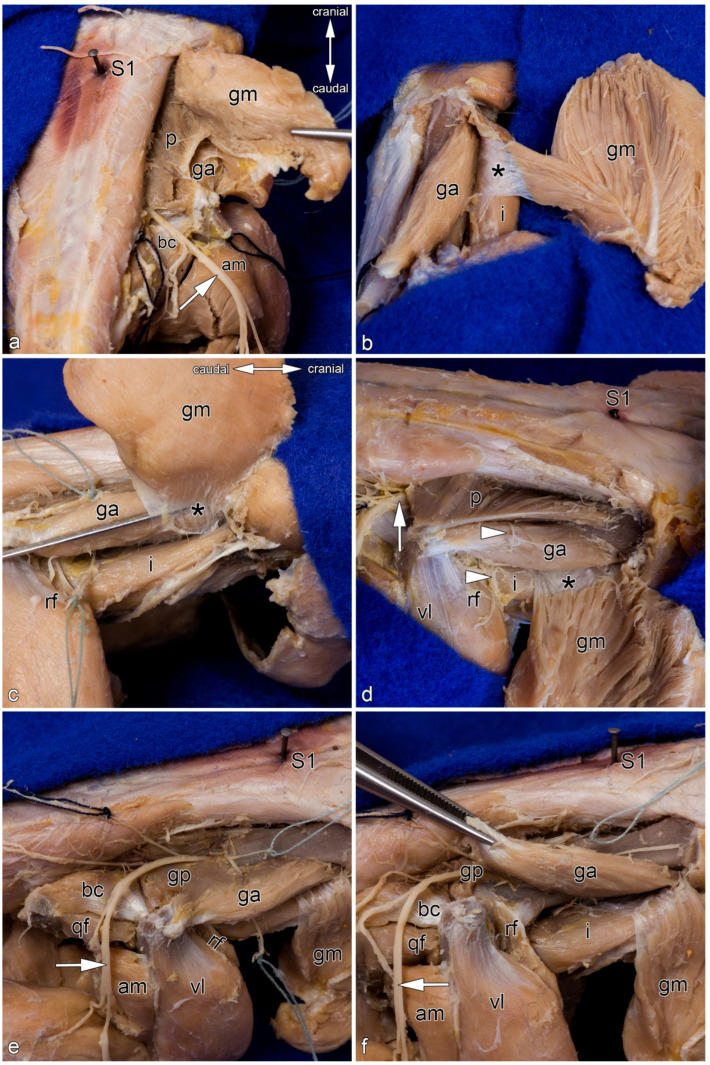
Topography of the gluteal muscles 2. (**a**) Dorsocaudal aspect. Separation of the gluteus medius muscle from the piriformis muscle, starting from its insertion. (**b**) Origin of the gluteus medius muscle from an aponeurotic intermuscular septum (asterisk). (**c**) Details of this intermuscular septum from the lateral aspect. (**d**) Dorsal view. At the craniolateral border of the piriformis muscle, the cranial gluteal nerve (white arrowheads) and at its caudal border, the caudal gluteal and sciatic nerves (white arrow) appear. (**e**) Dorsolateral view. The cranial gluteal nerve (light blue yarn) gives off the innervation for the gluteus medius muscle (cut through) before it pierces the gluteus accessorius muscle. The caudal gluteal nerve (dark blue yarn) originates deep to the piriformis muscle (already removed) from the sciatic nerve (white arrow). (**f**) The insertions of the gluteus accessorius and partly of the gluteus profundus muscles have been detached to show their relationship to the origin of the two tendons of the rectus femoris muscle. For details refer to the text. White arrow = sciatic nerve, am = adductor minimus and magnus muscles, bc = biceps coxae muscle, ga = gluteus accessorius muscle, gm = gluteus medius muscle, gp = gluteus profundus muscle, i = iliacus muscle, p = piriformis muscle, qf = quadratus femoris muscle, rf = rectus femoris muscle, S1 = spinous process of the first sacral vertebra, vl = vastus lateralis muscle.

The gluteus medius muscle covered all further muscles of the gluteal region. To reach the layer deep to it, its caudal tongue-like portion was released from the insertion on the proximal aspect of the third trochanter (Figure 13d). This tendon was also in close contact to the common tendon of the biceps coxae muscle, the sciatic nerve, and the caudal gluteal nerve. Subsequently the gluteus medius muscle was released from the spinous processes of the first to third sacral vertebrae (Figure 14a). In doing so a fine nerve twig from the caudal gluteal nerve, which innervated the most caudal portion of the gluteus medius muscle, was stretched (Figure 6d) and had to be cut through. The origin from the fascia of the muscles of the tail got visible (Figure 6b) and was released too. Separating the gluteus medius muscle from the deeper situated piriformis muscle was somewhat challenging, as there was no distinct fascia or natural gap between them. It was done best starting at their tendons on the greater trochanter. There, the gluteus medius muscle inserted on the caudolateral aspect, covering the tendon of the gluteus accessorius muscle, which was attached on the craniolateral aspect of the greater trochanter. By carefully elevating the gluteus medius muscle, its tendon could be released without harming the gluteus accessorius muscle (Figure 13d). Near the insertion of the gluteus medius muscle, attention also had to be paid to the origin of the vastus lateralis muscle. During releasing the tendon of the gluteus medius muscle, the tendon of the piriformis muscle, which inserted on the tip of the greater trochanter, became visible. The gluteus medius muscle was then reflected cranially by detaching it from the tendon of the piriformis muscle, without harming the latter. (Figure 14a). Close to the greater sciatic notch, in a gap between the piriformis and gluteus accessorius muscles, a branch from the cranial gluteal nerve appeared. It divided into several twigs, which entered the deep aspect of the gluteus medius muscle (Figure 6c). The nerve was cut through and the origin of the gluteus medius muscle from the gluteal surface between the dorsal and ventral gluteal lines was detached by blunt dissection. The whole muscle was reflected laterally (Figure 14b). Now its deepest portion could be completely observed. It originated by an aponeurosis, which was attached to the ilium between the ventral cranial iliac spine and the spina alaris. This aponeurosis encompassed the gluteus accessorius muscle completely and separated the latter from the iliacus muscle forming an intermuscular septum between their cranial portions. Caudally, the two muscles were separated only by their own fasciae, allowing the cranial gluteal nerve to pass through to reach the tensor fasciae latae muscle. Both, the gluteus accessorius and iliacus muscles originated partly from this septum (Figure 14c). As described above, the deep origin of the tensor fasciae latae muscle was attached to its ventrolateral aspect.

After reflecting the gluteus medius muscle, the piriformis and gluteus accessorius muscles became entirely visible. The latter was partly covered by the long feather-shaped piriformis muscle. At the craniolateral border of the piriformis muscle, the nerve to the gluteus medius muscle from the cranial gluteal nerve appeared. The exit of the cranial gluteal nerve through the greater sciatic notch was still partially covered by the piriformis muscle. At the caudal border of the piriformis muscle, the sciatic and caudal gluteal nerves appeared (Figure 14d).

The piriformis muscle was first detached from its insertion on the tip of the greater trochanter. Here, its tendon was in close contact with all the other tendons attaching to the greater trochanter. The gluteus medius and gluteus accessorius muscles inserted on the lateral aspect, the tendon of the gluteus profundus and biceps coxae muscles inserted on the medial aspect. With its proximal origin from the lateral aspect of the greater trochanter, the vastus lateralis muscle was also close to the insertion of the piriformis muscle. By lifting the distal portion of the piriformis muscle, one to three fine nerve twigs became visible, entering its deep surface (Figure 6f). These nerves were cut through, and the origin of the muscle was detached. This exposed the gluteus profundus muscle and the entire course of the sciatic and cranial gluteal nerves as they appeared in the greater sciatic notch. Still covered by the piriformis muscle and superficial to the gluteus profundus muscle, the caudal gluteal nerve and the nerve to quadratus femoris muscle arose from the sciatic nerve (Figure 14e). The cranial gluteal nerve coursed in a craniolateral direction and gave off the branch for the gluteus medius muscle. Then it coursed to the ventral aspect of the gluteus accessorius muscle to reach the gap between the latter and the iliacus muscle. It could not be clearly determined whether it passed through the gluteus accessorius muscle or coursed between this muscle and the gluteus profundus muscle. In any case, it sent branches to both. Its terminal branches innervated the tensor fasciae latae muscle as described above. Cranial to this passage, the gluteus accessorius and iliacus muscles were separated by the intermuscular septum formed by the deep aponeurotic origin of the gluteus medius muscle (Figure 14e). Near their insertion, the gluteus accessorius and profundus muscles covered the two heads of origin of the rectus femoris muscle. In addition, on the ventrolateral aspect of the greater trochanter, the tendon of the gluteus accessorius muscle was in close contact to the origin of the vastus lateralis muscle. The gluteus profundus muscle inserted near the tendon of the biceps coxae muscle. It covered the cranial portion of the gemellus muscle (Figure 14f). The mobilization from the latter was done by careful blunt dissection along the nerve to quadratus femoris muscle. This nerve coursed between these two muscles to enter the deep aspect of the biceps coxae muscle. Before it disappeared, it gave off a branch for the most caudal fibres of the gluteus profundus muscle (Figure 7b).

The gluteus accessorius muscle encompassed the cranial portion of the gluteus profundus muscle. No natural gap or fascia between these two muscles could be used to separate them in situ (Figure 14e). It was only possible to separate them once they were removed completely. Therefore, the gluteus profundus and accessorius muscles were detached from their insertion on the greater trochanter. The gluteus profundus muscle was in addition carefully peeled off from the capsule of the hip joint. Then, both muscles were released by blunt dissection towards their origin and removed as a whole. On their deep aspect a fine gap was visible, along which they could be separated without harming them (Figure 7a).

## 4. Discussion

### 4.1. Muscles of the Gluteal Group

#### 4.1.1. Superficial Portion (Gluteus Superficialis, Tensor Fasciae Latae, Femorococcygeus Muscles)

In mammals, the gluteus superficialis, tensor fasciae latae and femorococcygeus muscles are usually located in the most superficial layer of the gluteal muscles. Often, they are even fused forming a single continuous plate, with the femorococcygeus muscle most commonly appearing as a separate structure [1,8,9]. Also, in (anthropoid) apes, domestic animals and rodents, it is commonly reported that the gluteus superficialis and tensor fasciae latae muscles are usually fused [5,12,15,17,33], forming a kind of fleshy V, which is based on an aponeurosis [1]—in human anatomy called gluteal aponeurosis [32]. Essentially, this description also applies to human anatomy. Frohse and Fränkel [2] have already argued that the tensor fasciae latae muscle should more accurately be called *M. gluteus anterior*, and the gluteus maximus muscle should be referred to as *M. gluteus posterior*. According to these authors, a tendinous component originates between these two muscular parts, mainly from the iliac tubercle. All three parts together form the iliotibial tract [2], which is primarily attached to the lateral condyle of the tibia and also contributes to the lateral patellar retinaculum [2,4,34]. As the current study shows, this is likely also valid for albino rats. Unlike in the rats studied here, in humans the iliotibial tract also extends to the head of the fibula [4,34]. As we have shown in the current study, the iliotibial tract is considerably weaker in rats than in humans. In some quadrupeds, it is even entirely absent; in such cases, the gluteus superficialis and tensor fasciae latae muscles both attach directly to the third trochanter [35]. The stronger iliotibial tract in humans is likely due to the bipedal gait and the associated extension of the hip and knee joints, as well as the elongation of the femur. This upright posture obviously requires greater support [36]—often described as tension band effect [37,38]—compared to that of quadrupeds. This factor may possibly led to the development of a substantially stronger gluteus maximus muscle in humans compared to the gluteus superficialis muscle found in most mammals [1,5,17,39]—including the rats studied here.

It appears that the gluteus maximus muscle in humans is also ‘more’ than the gluteus superficialis muscle in animals [9]. If one compares the findings presented here regarding the femorococcygeus and gluteus medius muscles, one gets the impression that both, the femorococcygeus muscle and the deep, tongue-shaped portion of the gluteus medius muscle have been ‘incorporated’ into the human gluteus maximus muscle. The facts that both are already innervated by the caudal gluteal nerve in rats, that the deep portion of the gluteus medius muscle inserted on the third trochanter, and that the femorococcygeus muscle already reinforced the iliotibial tract may be indications for this idea. Furthermore, it has already been shown that the human gluteus maximus muscle develops embryonal from two parts, the more distally located of which is thought to correspond more or less to the femorococcygeus muscle [40]. The femorococcygeus muscle is also referred to as the agitator caudae muscle [9], which apparently highlights its main function—the movement of the tail. For us, this again could explain why, in species with a reduced tail (e.g., humans versus rats), it seems to atrophy and to fuse to different degrees with neighbouring muscles. In humans occasionally, an additional bundle from the gluteus maximus muscle has been observed. This ‘caudal head’ originates from the coccyx and inserts on the femur [39,41,42]. Furthermore, fascicles of the gluteus maximus muscle can extend considerably further distally before they are attached to the iliotibial tract [39]. Both these variants may be the remnant of a femorococcygeus muscle, which is occasionally not completely fused with the gluteus maximus muscle.

Previous authors dealing with comparative anatomy, or the anatomy of rodents have described the origin of the gluteus superficialis and tensor fasciae latae muscles in a similar way to what has been observed here. Thus, the tensor fasciae latae muscle originates variably from the iliac crest, the caudal lumbar and cranial sacral vertebrae, and from a fascia between the gluteus medius and iliacus muscles [1,5,15,17]. We regarded this fascia as an aponeurotic origin of a part of the gluteus medius muscle, forming a kind of an intermuscular septum (Figure 13c). Given that the wing of ilium is much wider and separating the gluteal muscles completely from the iliacus muscle in humans, this septum may have regressed in favour of the bone. What may remain in humans, is the deep attachment of the iliotibial tract around the anterior inferior iliac spine—so called *Tractus praetrochantericus* according to Frohse and Fränkle [2], also known as the ‘deep anchor’ of the iliotibial tract [43]. The gluteus superficialis muscle is said to originate variably from the sacrum (or the sacral vertebrae), the cranial coccygeal vertebrae, and the gluteal aponeurosis. It inserts partly on the third trochanter. The remaining portion forms together with the tensor fasciae latae muscle the iliotibial tract [1,5,15,17]—as discussed above. All these findings are consistent with the results obtained here. In addition, we observed, that the deep portion of the iliotibial tract was attached to the full length of the lateral lip of the facies aspera, thus forming the lateral intermuscular femoral septum, like in humans [1,2]. Although the tensor fasciae latae muscle formed a common musculoaponeurotic plate with the gluteus superficialis muscle in the here examined animals, it was—in contrast to the gluteus superficialis muscle—innervated by the cranial gluteal nerve, like that observed in other mammals [1,9,16]. This, too, can be explained by embryogenesis. The tensor fasciae latae muscle is derived from the cranial aspect of the deep gluteal mass, but soon separates from it, fusing with the superficial gluteal muscle to extend distally over the vastus lateralis muscle in the thigh. The two muscles can only be distinguished by their points of origin and their different innervation [6]. We, too, had no other way of distinguishing these two muscles. In addition to purely macroscopic observation, we tried to define the innervation areas of the two gluteal nerves more precisely using the modified Sihler’s procedure (Figure 4f). Our results indicate that the two nerves apparently anastomosed within the musculoaponeurotic plate. This may be a further hint of the complete fusion of both muscles, even though, as described above, they originally develop from two distinct anlagen.

The femorococcygeus muscle is said to arise from the spinous process of the last sacral and/or first coccygeal vertebrae and to insert on the ’lateral side of the femur’, the lateral femoral condyle, the ‘crest’ of the tibia, the capsule of the knee joint or even the patella [1,5,15,17,35]. In our cases, it arose only from the first coccygeal vertebra—partly covered by the semitendinosus muscle. It reinforced the lateral patellar retinaculum and the iliotibial tract. With its deep portion it was attached to the lateral femoral epicondyle and the lateral fabella, both conjoined with part of the biceps femoris muscle. Due to its close relationship to the latter, the femorococcygeus muscle is often referred to as gluteobiceps muscle [14,32], or caudal (or cranial/anterior) head of biceps femoris [10,11,44]. Such apparent misinterpretations have already been criticized by Appleton [8]. It also becomes apparent at an early stage of the mouse’s development that the femorococcygeus muscle splits off caudally and the gluteus maximus (superficialis) muscle cranially from the superficial gluteal mass [6]. In the current study, the muscle referred to here as the femorococcygeus muscle was identified as such and as part of the superficial gluteal muscle group, first and foremost by its innervation by the caudal gluteal nerve, but also by its topographical features described earlier [1,6,8,9,15,16,17,19].

Some authors also mentioned the occasional presence of a tenuissimus muscle within the superficial gluteal muscles in some mammals. This is thought to correspond to the iliofibularis muscle of reptiles [9,45]. It originates either from the sacral and/or coccygeal vertebrae, or the gluteal aponeurosis, and inserts on the ‘lateral side of the lower leg’ [1,9] or on the tendo calcaneus communis [35]. It is sometimes confused with the femorococcygeus muscle but is situated deep to the superficial gluteal muscles. While the femorococcygeus muscle is innervated by the caudal gluteal nerve, the tenuissimus muscle is innervated by the dorsal sciatic nerve (=common peroneal nerve) [1]. As it often unites with the biceps femoris muscle [9], it is assumed that the short head of the biceps femoris muscle of higher primates including humans might derive from the tenuissimus muscle [1,9,45]. In rodents, it is said to blend distally with the biceps femoris muscle to form its second head (caput coccygeus) [1], but it is not always present [15,16]. Its anlage was observed in the early stages of development in mice, however it soon vanished [6]. The presence of such a muscle was not observed in any of the rats examined in this study Therefore, we are unable to comment further.

#### 4.1.2. Deep Portion (Gluteus Medius, Piriformis, Gluteus Profundus, Gluteus Accessorius Muscles)

The deeper muscles of the gluteal group show different divisions and fusions [9,17,35,39,46]. Through all species including humans, they all insert on or around the greater trochanter [1,2,4,5,9,36,47].

The piriformis muscle is often seen as a splitting off from the gluteus medius muscle [6,9]. These two muscles are therefore generally—also in rodents—not always clearly separable [1,9,14,15,48]. This fact can also be attributed to the embryogenesis of these muscles. The deep gluteal mass (innervated by the cranial gluteal nerve) initially divides into three parts: the anlage for the tensor fasciae latae muscle (which subsequently migrates superficially as described above, whilst retaining its innervation by the cranial gluteal nerve), the anlage for the gluteus medius muscle, and the anlage for the gluteus minimus (profundus) muscle. The gluteus medius muscle has a two-layered origin, arising not only from the gluteal surface of the ilium, the iliac crest, and the cranial part of the ‘inferior margin of the ilium’, but also from the spine caudal to the second sacral segment. From this, a slip splits off caudodorsally, which gives rise to the piriformis muscle [6]. It is possible that this division may not always occur in the same way, which could explain the various appearances and areas of origin of the piriformis muscle across species. On the other hand, it could also be that this division sometimes does not or only partially occur, which might explain why the two muscles cannot always be clearly distinguished. Although we initially encountered difficulties in separating these two muscles in a reproducible manner during our first dissection attempts, we were finally able to do so. To achieve this, we had to accept that—unlike in humans or other mammals [1,4,5,17,34]—the piriformis muscle had a broad area of origin on the gluteal surface of the ilium between the dorsal and ventral gluteal lines, described similarly only in exceptional cases for rodents [15]. We were able to consistently separate the gluteus medius muscle along the long tendon of the piriformis muscle, even though there was no visible intermuscular fascia or natural gap at this point. Only the nerve branches entering the piriformis muscle coursed within this layer. Some authors have observed that, in humans, the piriformis muscle may also be fused to varying degrees with the gluteus medius muscle or its tendon [39,42,49,50]. This, in turn, may indicate their common ancestry and varying separation.

The gluteus medius muscle is usually the largest of the gluteal muscles in mammals [1,5,12,14], which was also the case here. Also, several parts are often described in mammals [1,14,16] including humans [39,46,47,51,52]. The rats examined here exhibited a caudal, tongue-shaped portion that was clearly separated from the rest of the muscle towards its insertion. Unlike the rest of the muscle, this portion was attached on the third trochanter and was innervated by the caudal gluteal nerve. A superficial portion of the gluteus medius muscle of domestic animals is said to join with the gluteus superficialis muscle to form the gluteus maximus muscle of humans [5]. It is possible that this could also be true for this tongue-shaped portion as we have already discussed above.

The gluteus medius muscle is said to have a large area of origin, which is described very variably. Among others, it originates from the gluteal surface of the ilium, an aponeurotic sheet arising from the “ventral margin of the ilium”, from the iliac crest, and from the spinous processes of lumbar and sacral vertebrae [1,11,14,15,16,17]. Based on our results, we were able to confirm all these findings for the albino rat.

The piriformis muscle arises either only from the sacrum [5,10,14,16,17] or also from parts of the ilium [1,13,15]. The latter was also true in our cases. It even occupied a relatively large area between the dorsal and ventral gluteal lines, immediately adjacent to the gluteus medius muscle. This broad field of origin from the ilium is probably due to the fact, that the wing of ilium is considerably longer in rats than in humans. In the latter, the wing of ilium is wider but shorter, so that the gluteus medius muscle occupies the entire area between the posterior and anterior gluteal lines [4,34]. Therefor it could be possible, that the origin of the piriformis muscle becomes limited towards the greater sciatic notch, the sacrotuberous ligament, and the sacrum, as described for humans [4,34,39,47,50].

The gluteus profundus muscle is usually completely enveloped by the gluteus medius muscle and is therefore not always easily distinguishable from the latter [1,13]. We did not have any trouble distinguishing the gluteus profundus muscle from the gluteus medius or piriformis muscles. We actually had more trouble distinguishing it from the much less commonly and heterogeneously described gluteus accessorius (scansorius) muscle [53]. From the superficial aspect, the two muscles appeared to be a single entity. After several attempts to separate them along the cranial gluteal nerve, amongst other methods, we concluded that it was better to separate the muscles after harvesting them in one part. Only then did the small gap between them (Figure 7a) become visible—and thus accessible. The gluteus accessorius muscle is described as a ventral division of the gluteus profundus muscle and is said to be common in rodents [1,6,9,12,13]. In the rats examined here, it was noticeably the larger of the two muscles. This is, again, probably due to the elongated wing of ilium in the rat. In addition, in some rodents, the gluteus profundus muscle is not clearly distinguishable from the cranial gemellus muscle [16]. In the animals examined here too, it had a close relationship with the gemellus muscle. In addition, its caudal portion also had the same innervation; namely a branch of the nerve to quadratus femoris muscle. As we reported recently [25], the albino rat does not have a superior gemellus muscle. It may be fused with the gluteus profundus muscle (leading to its double innervation) but separated from the obturator internus and gemellus muscles. However, this would have to be a secondary process, as the two muscles are from different derivatives (dorsal versus ventral sacral mass) [6].

In humans, it is likely that—at least in most cases—a single muscle, namely the gluteus minimus muscle, develops. However, muscles corresponding to a gluteus accessorius (or gluteus quartus or scansorius) muscle have also been observed in hominids and humans. However, the descriptions here are also very heterogeneous [12,39,41,46,53,54]. Other authors generally described the gluteus minimus muscle as consisting of two or more parts [3,4,34,47,52,55]. According to our own observations and some former authors [42,46], in humans there are occasionally accessory muscle bundles covering the anterior portion of the gluteus minimus muscle. These bundles are situated superficial to the course of the superior gluteal nerve—just like the gluteus accessorius muscle observed in the present study. These earlier observations might suggest that the gluteus accessorius muscle may also be present, at least in a rudimentary form, in humans.

The gluteus profundus muscle arises variably from the gluteal surface and other parts of the ilium, up to the greater sciatic notch and the ischial spine. Its insertion is usually described as being on and around the greater trochanter [1,11,14,15,16,17]. This is likely to be very consistent with only small variations across all species including the albino rat we examined. Although the gluteus profundus muscle is regarded as the analogue of the human gluteus minimus muscle, in our cases it inserted on the medial aspect of the greater trochanter (like the triceps coxae muscle in humans [3,4,34]), whilst the gluteus accessorius muscle inserted craniolaterally on the greater trochanter (like the gluteus minimus in humans [4,34,52,55]). In humans, the gluteus minimus muscle also reinforces the hip joint capsule [4,34,52,56]. In the rats studied here, this was the case only for the gluteus profundus muscle.

### 4.2. Muscles of the Hamstring Group

#### 4.2.1. Semitendinosus Muscle

In many animals, the semitendinosus muscle consists of two heads. The so-called vertebral head originates from the spinous processes of the last sacral and first coccygeal vertebrae. The pelvic (or ischial) head usually arises together with the biceps femoris muscle from the ischial tuber [1,9]. Sometimes the two heads even form completely separate muscles [35,46]. In domestic animals, the vertebral head is only described for horses and pigs [5,14]. In anthropoids, this additional head is apparently no longer present [12]. Two heads of origin are also described for most rodents [11,13,16,17], including the albino rat [10]. This was verified with the result of the present study. The two heads of the semitendinosus muscle were always present and showed constant areas of origin, i.e., from the spinous processes of the first and second coccygeal vertebrae, and the dorsocaudal aspect of the ischial tuber respectively. Usually, the two heads fuse, often forming a tendinous intersection at this point [1,5,9,12,57]. This had also been reported for some rodents [16,17]. Also in the present study, the two heads united forming a tendinous intersection to which the biceps femoris muscle was also attached, which is described only exceptionally [1,16]. Although the vertebral head—at least in this form—is no longer present in humans, this tendinous junction is still present in the proximal portion of the muscle [2,3,4,34,42]. As described above, the biceps femoris muscle was also attached to this tendinous intersection present in the here examined albino rats. We think that this may be the precursor for the common head formed by the semitendinosus muscle and the long head of the biceps femoris muscle arising from the ischial tuber found in humans [4,34,39].

In the present study, the two heads of the semitendinosus muscle were innervated by two different sources—probably due to topographical relationship. The nerve branch supplying the vertebral head coursed with the superficial perineal nerve, whilst the remaining muscle was innervated by the hamstring muscular branch. However, the branch for the pelvic head entered the muscle distal to the tendinous intersection (Figure 8a). Evidence that the nerve innervating the vertebral head was the superficial perineal nerve was based on its topographical relationship with this part of the muscle and with the caudofemoralis muscle, as described earlier [1,6,19], and its origin from the pudendal nerve [29]. In humans, too, different nerve branches—both from the tibial nerve—enter the muscle both proximally and distally to the tendinous intersection [2,4,34]. This fact for us indicates that the vertebral head of the semitendinosus muscle, which is present in many quadrupeds, may be still present in humans as well.

The distal tendon of the semitendinosus muscle often fuses with that of the sartorius and gracilis muscles, and with the deep fascia of leg [1,5,11]. In some species the insertion extends far distally, in ruminants even as far as the calcaneus [1,14]. In many rodents the muscle also inserts medially on the tibia [13,15,16,17], which was also true in the present study. Although there was a close relationship between the tendons of the semitendinosus and caudal gracilis muscles in the rats examined here, there were no connections between these two tendons; a sartorius muscle was never present. Compared with the rats examined here, the tendon of the semitendinosus muscle in humans is notably elongated, but its insertion occurs at a similar position, namely on the proximal portion of the medial surface of the tibia [4,34]. As discussed above for the distal migration of the pelvic head, the elongated distal tendon might also have been caused by the extension of the hip and knee joints, as well as the elongation of the femur in humans [11].

#### 4.2.2. Biceps Femoris Muscle

In animals, the biceps femoris muscle is described very heterogeneously. According to some authors, it consists of two heads. One originates from the ischial tuber, and the second from the sacrum or the cranial coccygeal vertebrae [5,14]. However, confusion with other muscles is likely to occur. The proper biceps femoris originates in all species exclusively from the ischial tuber [1,9], and is likely to correspond solely to its long head in humans [1]. In some specimens an additional head is described [10,11,44]. This seemingly corresponds to the femorococcygeus muscle; however, the latter belongs to the gluteal group [1,6,8]. As the femorococcygeus muscle is said to partially fuse with the biceps femoris in some species, the term gluteobiceps muscle was also introduced as an official term [1,5,29,33]. In most rodents, the biceps femoris muscle is described as a singular structure, originating only from the ischial tuber [15,16,17]. Rarely, two heads are also described in rodents [11], including the albino rat [10]. However, our results show that the biceps femoris muscle was a singular structure in the albino rat. It was clearly separated from the femorococcygeus muscle by the caudal cutaneous femoral nerve [19], and the lateral cutaneous sural nerve [15].

The rarely described so-called femoral or short head (like in humans and higher primates) seems to be rather derived from the tenuissimus muscle rarely found in mammals than from the femorococcygeus muscle [1,9,45], or is even regarded as a new acquisition [8]. However, the origin of the short head has not yet been fully clarified.

The biceps femoris muscle usually forms a broad tendon of insertion being situated on the lateral side of the lower leg. The cranial border of the tibia, the deep fascia of leg, and occasionally the patella or even the calcaneus are named as attachments [5,9,11,14]. In anthropoids, the biceps femoris muscle also inserts on the fibular head [12], like in humans [4,34]. In most rodents, the biceps femoris muscle inserts lateral on the proximal two-thirds of the tibia via the deep fascia of leg [15,16,17]. In the present study, it inserted via a broad aponeurosis on the proximal half to two thirds of the cranial border of the tibia. In addition, a deep portion was attached to the lateral femoral epicondyle (which was given as point of insertion only exceptionally [10]), and to the lateral fabella, which has not been described for rodents until now. This deep portion was conjoined with the femorococcygeus muscle, which may have led some authors to regard the latter as an additional head of the biceps femoris muscle [5,10,11,13,14,58]. Even though the main insertion of the biceps femoris muscle in humans has shifted to the head of the fibula [9], its insertion on the tibia is still present. Namely, it attaches via an aponeurosis on the lateral tibial condyle, thereby reinforcing the knee joint capsule in the dorsolateral region [4,34,39,59,60]. Both, the insertion on the lateral fabella, and the aponeurotic connection to the lateral head of the gastrocnemius muscle described the first time in the present study, may provide an explanation for the occasional occurrence of connections between the biceps femoris and gastrocnemius muscles in humans [41].

#### 4.2.3. Semimembranosus and Caudofemoralis Muscles

The semimembranosus muscle is described as two-headed or doubled for some species [5,11,13,14], originating with one head from the ischial tuber and with the second from the first coccygeal vertebra [5,11], or in the immediate proximity of the first head [13]. It can also rarely occur duplicated in humans [39,42,46].

In many rodents it is described as a singular muscle arising in the region of the ischial tuber deep to the semitendinosus and biceps femoris muscles and inserting medially on the tibia [15,16,17]. In the rats examined here, too, it was a single structure. However, it did not merely attach medially to the tibia; rather, it divided distally into two parts that surrounded the tibial collateral ligament. In doing so, it reinforced the medial patellar retinaculum and, by attaching to the medial fabella, the knee joint capsule. In humans, too, it reinforces the knee joint capsule, primarily through its attachment to the dorsal aspect of the capsule forming the oblique popliteal ligament [4,34].

Additional muscles in proximity to the semimembranosus muscle are the caudofemoralis and praesemimembranosus muscles. The caudofemoralis muscle probably corresponds to the retractor dorsalis muscle of lower vertebrates [9]. Although it is supposed to be a primitive element, it is nevertheless present in many mammals [1,8,35]. For numerous rodents, however, the occurrence of a caudofemoralis muscles is always described [15,16,17]. It originates from the transverse processes of the cranial coccygeal vertebrae, or the ischial tuber, and inserts on the dorsomedial surface of the femur [1,15,16,17,35]. In the rats examined here, too, the caudofemoralis muscle originated from the spinous process of the first coccygeal vertebra. It inserted not only medially on the femur but also on the medial fabella close to the medial head of the gastrocnemius muscle. To our knowledge, the attachment on the medial fabella has not been described yet. However, its proximity to the medial head of the gastrocnemius muscle was mentioned [16].

The praesemimembranosus muscle is sometimes described as a splitting off of the semimembranosus muscle [9] or as an individual muscle, which originates in close proximity to the semimembranosus muscle and inserts on the medial femoral condyle [1]. During mouse embryogenesis, the anlage of a praesemimembranosus muscle exists for a short time, but subsequently disappeared [6]. In none of the rats examined here was a praesemimembranosus muscle present; although, we briefly considered this at the beginning of our investigation. Thus, we attempted to separate the semimembranosus muscle starting from its bifurcated insertion towards its origin. However, we were unable to achieve this in a reproducible manner. This raises the question of whether this distal division might correspond to a rudimentary praesemimembranosus muscle or not.

Due to the close anatomical proximity between the praesemimembranosus and caudofemoralis muscles, confusion or problems in their definition are likely to occur repeatedly. As they only occur together in exceptional cases, they are often confused with one another or not recognized as such at all. Occasionally, the femorococcygeus muscle is even referred to as the caudofemoralis muscle [1,8,19]. The information provided by different authors is often difficult or impossible to compare if, when naming a muscle, no details are given regarding its innervation or its topographical relationship to nerves, as often done [10,13,35,44,58,61]. In addition, these two muscles are apparently not known to many authors, probably because they are not defined in the *Nomina Anatomica Veterinaria* [29]. They are therefore probably often described as parts of other muscles (e.g., the semimembranosus muscle as mentioned above) [5,10,11,13,14].

The caudofemoralis muscle is located (deep) ventromedially to the tenuissimus muscle (if present) and to the sciatic nerve, but superficially (dorsolaterally) to the hamstring branch. If it originates from the vertebral column, it is also situated superficial to the pudendal and superficial perineal nerves and the nerve to quadratus femoris muscle. The praesemimembranosus muscle is located deep (medially) to the hamstring muscular branch and, consequently, when both are present, also deep (medially) to the caudofemoralis muscle [1,8,19].

We were able to clearly identify the muscle consistently present in our rats as the caudofemoralis muscle, based on previous descriptions mentioned above [1,6,8,15,16,19]. As we recently discussed [24], given its location, innervation and topographical relationship to the adductor hiatus, the caudofemoralis muscle seems to fuse with the adductor magnus muscle in humans forming its ischiocondylar portion. However, there is also a view that, in primates and humans, the praesemimembranosus muscle is thought to merge with the adductor magnus to form its ischiocondylar portion [1,61].

### 4.3. Sacrotuberous Ligament

As recently reported, the examined albino rats lacked a sacrotuberous ligament [25]. On the other hand, as observed in the present study, parts of the muscles of the gluteal (femorococcygeus muscle) and hamstring groups (semitendinosus and caudofemoralis muscles) originated from the coccygeal vertebrae. We think, this is most likely due to the prominent and mobile tail. In humans, the almost complete loss of the tail, the extension of the hip and knee joints, and above all the development of bipedal locomotion changed the demands on the skeleton, ligaments and muscles, which has already been discussed earlier [11]. Muscles that previously moved both the hip and knee joints as well as the tail seem to lose the latter function. As a result, they might have also lost their attachment to the vertebral column—perhaps only apparently so. It is possible that the muscle belly ‘slips’ caudally, and the origins from the ischial tuber become more prominent. This may result in the formation of the sacrotuberous ligament in the characteristic form of humans. Previous authors have also put forward similar theories [8,62], although the origin of the sacrotuberous ligament remains unclear. Recent studies also show that the tendons of origin of the hamstring muscles and the ischiocondylar portion of the adductor magnus muscle are closely associated with the sacrotuberous ligament, and that the latter has a multi-layered structure [63,64]. According to our own observations in human specimens, these tendons extend in part over the ischial tuberosity and continue proximally into the ligament.

### 4.4. Limitations

Due to availability, this study is based only on male rats. Although we do not expect any differences regarding topography, our results must therefore be verified in female rats.

We have described the nerves relevant to this study in detail only in the periphery. The study of the formation of the sacral plexus and possible variations was not set as an aim. It must therefore be investigated in the albino rat in detail in further studies.

The apparent double innervation of the gluteus profundus muscle was not investigated in more detail in this study. Further investigation is also required here to determine whether, and to what extent, the branch from the nerve to quadratus femoris muscle contributes to the innervation of this muscle.

We were only able to address and discuss earlier authors’ theories on phylogeny of the muscles of the gluteal and hamstring groups on the basis of observations of only one single species. Furthermore, our comments have mainly focused on the comparison between albino rats and humans, as our current practical experience is based on these two species. Further research involving a wide range of species is needed to gain a better understanding of the muscle groups examined here.

## 5. Conclusions

The albino rats examined here presented a consistent pattern for the muscles of the gluteal and hamstring groups. The individual muscles could be clearly identified based on their topography and innervation. Within the gluteal group, the gluteus superficialis and tensor fasciae latae muscles formed an inseparable musculoaponeurotic plate. The femorococcygeus muscle was situated caudally to this. Although the gluteus medius and piriformis muscles were closely related, they could always be separated. In addition to the gluteus profundus muscle, there was also always a gluteus accessorius muscle present. Of the muscles belonging to the hamstring group, the two-headed semitendinosus, the one-headed biceps femoris, the semimembranosus, and the caudofemoralis muscles were always present.

These findings make a valuable contribution to understand the topographical anatomy of the muscles and nerves of the gluteal and hamstring groups in the albino rat. In future studies using rats as animal models (e.g., harvesting distinct muscles or experimental surgery), it should now be possible to clearly identify and name all the muscles and nerves within these muscle groups. This study also provides a basis for further comparing these muscle groups with other species, including humans.

## Figures and Tables

**Table 1:** Systematic anatomy of the muscles of the gluteal group in the albino rat. For each muscle, the exact origin, insertion, and innervating nerves are summarized here. Within this muscle group, there were only rare remarkable variations, concerning only the course of the innervation.

Muscle	Origin	Insertion	Innervation
Gluteus superficialis and tensor fasciae latae muscles	Both, including gluteal aponeurosis: Spinous processes of all four sacral and the first coccygeal vertebrae (aponeurotic). Tensor fasciae latae muscle: Ventral cranial iliac spine, iliac crest, dorsal cranial iliac spine Aponeurotic septum between gluteal muscles and iliacus muscle (=deep origin of gluteus medius muscle)	Gluteus superficialis muscle: Cranial aspect of third trochanter. both: Forming iliotibial tract. Lateral femoral and lateral tibial condyles (reinforcing lateral patellar retinaculum). Lateral lip of facies aspera (forming lateral femoral intermuscular septum).	Caudal gluteal nerve (gluteus superficialis muscle). Cranial gluteal nerve (tensor fasciae latae muscle).
Femorococcygeus muscle	Spinous process of the first coccygeal vertebra (together with the caudofemoralis muscle).	Lateral patellar retinaculum. Lateral femoral epicondyle and lateral fabella (conjoined with biceps femoris muscle).	Caudal gluteal nerve. Direct branch from sacral plexus (exceptional).
Gluteus medius muscle	Spinous processes of first to third sacral vertebrae. Deep aspect of gluteal aponeurosis. Fascia covering the dorsal muscles of tail. Iliac crest (deep to tensor faciae latae muscle). Gluteal surface of ilium between dorsal and ventral gluteal lines (cranial to piriformis muscle). Via an aponeurotic septum from ventral cranial iliac spine and spina alaris (situated between gluteal muscles and iliacus muscle).	Caudolateral aspect of greater trochanter. Proximal portion of third trochanter.	Cranial gluteal nerve. Caudal gluteal nerve (most caudal portion of muscle).
Piriformis muscle	Caudal portion of lateral border of wing of sacrum and transverse process of third sacral vertebra. Gluteal surface of ilium between dorsal and ventral gluteal lines (caudal to gluteus medius muscle). Cranial portion of greater sciatic notch.	Tip of greater trochanter.	1–3 muscular branches from sciatic nerve (exceptional also from cranial gluteal nerve).
Gluteus accessorius muscle	Gluteal surface of ilium between ventral and caudal gluteal lines. Aponeurotic septum between gluteal muscles and iliacus muscle (=deep origin of gluteus medius muscle).	Craniolateral aspect of greater trochanter.	Cranial gluteal nerve.
Gluteus profundus muscle	From ischial spine and greater sciatic notch to dorsal border of acetabulum.	Medial aspect of greater trochanter (dorsocranial to biceps coxae muscle). Capsule of hip joint.	Cranial gluteal nerve. Nerve to quadratus femoris muscle (most caudal portion of muscle).

**Table 2:** Systematic anatomy of the muscles of the hamstring group in the albino rat. For each muscle, the exact origin, insertion, and innervating nerves are summarized here. Within this muscle group, there were only rare remarkable variations concerning only the course of the innervation.

Muscle	Origin	Insertion	Innervation
Semitendinosus muscle	Vertebral head: Spinous processes of the first and second coccygeal vertebrae (aponeurotic). Pelvic head: Dorsocaudal aspect of ischial tuber.	Cranial border of tibia (distal to cranial gracilis muscle).	Superficial perineal nerve or only exceptionally hamstring muscular branch from tibial nerve (vertebral head). Hamstring muscular branch from tibial nerve and only exceptionally in addition direct branches from tibial nerve (pelvic head).
Biceps femoris muscle	Dorsocranial aspect of ischial tuber.	Superficial portion: Lateral patellar retinaculum. Proximal half to two thirds of cranial border of tibia. deep portion: Lateral femoral epicondyle and lateral fabella (conjoined with femorococcygeus muscle).	Hamstring muscular branch from tibial nerve.
Semimembranosus muscle	Ventral aspect of ischial tuber. Caudal margin of tabula of ischium.	superficial portion: Medial patellar retinaculum. Medial tibial condyle and medial surface of tibia medial to patellar ligament. deep portion: Medial tibial condyle deep to tibial collateral ligament. Distomedial aspect of medial fabella.	Hamstring muscular branch of tibial nerve.
Caudofemoralis muscle	Spinous process of first coccygeal vertebra (together with femorococcygeus muscle).	Medial femoral epicondyle and medial supracondylar tuberosity. Proximomedial aspect of medial fabella.	Hamstring muscular branch or direct branch (exceptional) of tibial nerve. In addition (approx. half of limbs), from muscular branch for femorococcygeus muscle or vertebral head of semitendinosus muscle.

## Data Availability

Data is contained within this article.
